# Supplementary material for: The relationship between anemia and sleep disturbances among older Chinese adults: The mediating role of handgrip strength
Source: PLoS One. 2025 Oct 9;20(10):e0333673. doi: 10.1371/journal.pone.0333673 (PMC12510644; doi:10.1371/journal.pone.0333673)
Supplement: S1 Table — (DOC) [file pone.0333673.s001.doc]

S1 Table. Collinearity analysis result

| Term1 | VIF | DF | VIF^(1/(2*Df)) | colinearity |
| --- | --- | --- | --- | --- |
| Crude | 1.981 | 1 | 1.407 | 0 |
| Age | 1.287 | 1 | 1.134 | 0 |
| Sex | 3.129 | 1 | 1.769 | 0 |
| Residence | 1.083 | 1 | 1.041 | 0 |
| Marital status | 1.124 | 2 | 1.03 | 0 |
| Education Status | 1.12 | 1 | 1.058 | 0 |
| Smoking Status | 2.153 | 1 | 1.467 | 0 |
| Drinking Status | 1.24 | 2 | 1.055 | 0 |
| BMI | 1.143 | 3 | 1.023 | 0 |
| 14 chronic conditions | 1.032 | 2 | 1.008 | 0 |
| anemia | 1.055 | 1 | 1.027 | 0 |
| Abbreviations: VIF, variance inflation factors; DF, degree of freedom. A VIF of five or higher indicates multicollinearity | | | | |
