# Supplementary material for: The relationship between anemia and sleep disturbances among older Chinese adults: The mediating role of handgrip strength
Source: PLoS One. 2025 Oct 9;20(10):e0333673. doi: 10.1371/journal.pone.0333673 (PMC12510644; doi:10.1371/journal.pone.0333673)
Supplement: S2 Table — (DOC) [file pone.0333673.s002.doc]

| Variables | Total  (n = 9956) | Exclude participants  (n = 3899) | Included participants  (n = 6057) | p |
| --- | --- | --- | --- | --- |
| Age, Mean ± SD | 68.5 ± 7.1 | 69.8 ± 8.0 | 67.7 ± 6.2 | < 0.001 |
| Sex, n (%) |  |  |  | 0.031 |
| Female | 5040 (50.7) | 2020 (52.1) | 3020 (49.9) |  |
| Male | 4896 (49.3) | 1859 (47.9) | 3037 (50.1) |  |
| Residence, n (%) |  |  |  | < 0.001 |
| Rural | 5978 (60.1) | 2212 (57) | 3766 (62.2) |  |
| Urban | 3963 (39.9) | 1672 (43) | 2291 (37.8) |  |
| Marital status, n (%) |  |  |  | < 0.001 |
| Married and living with a spouse | 7455 (75.2) | 2729 (70.8) | 4726 (78) |  |
| Married but living without a spouse | 317 ( 3.2) | 174 (4.5) | 143 (2.4) |  |
| Single, divorced, and windowed | 2141 (21.6) | 953 (24.7) | 1188 (19.6) |  |
| Education Status, n (%) |  |  |  | 0.199 |
| Elementary school or below | 7877 (79.3) | 3047 (78.7) | 4830 (79.7) |  |
| Middle school or above | 2053 (20.7) | 826 (21.3) | 1227 (20.3) |  |
| Smoking Status, n (%) |  |  |  | 0.107 |
| Non-smoker | 5248 (53.2) | 2065 (54.2) | 3183 (52.6) |  |
| Smoker | 4618 (46.8) | 1744 (45.8) | 2874 (47.4) |  |
| Drinking Status, n (%) |  |  |  | < 0.001 |
| Drink but less than once a month | 726 ( 7.4) | 272 (7.2) | 454 (7.5) |  |
| Drink more than once a month | 2385 (24.3) | 833 (22.1) | 1552 (25.6) |  |
| Non-drinker | 6718 (68.3) | 2667 (70.7) | 4051 (66.9) |  |
| BMI group, n (%) |  |  |  | < 0.001 |
| Underweight | 660 ( 8.4) | 213 (11.3) | 447 (7.5) |  |
| Normal | 4769 (60.7) | 1180 (62.5) | 3589 (60.1) |  |
| Overweight | 2061 (26.2) | 424 (22.5) | 1637 (27.4) |  |
| Obesity | 372 ( 4.7) | 71 (3.8) | 301 (5) |  |
| Sleep duration(Hrs), Mean ± SD | 6.2 ± 2.1 | 6.2 ± 2.2 | 6.3 ± 2.1 | 0.615 |
| Daytime napping duration(Min), Median (IQR) | 40.8 ± 46.1 | 40.3 ± 46.0 | 41.0 ± 46.1 | 0.444 |
| 14 chronic conditions, n (%) |  |  |  | < 0.001 |
| 0 | 2013 (20.2) | 861 (22.2) | 1152 (19) |  |
| 1 | 2227 (22.4) | 874 (22.5) | 1353 (22.3) |  |
| ≥2 | 5701 (57.3) | 2149 (55.3) | 3552 (58.6) |  |
| Anemia, n (%) |  |  |  | 0.004 |
| Yes | 1461 (22.1) | 149 (27) | 1312 (21.7) |  |
| No | 5148 (77.9) | 403 (73) | 4745 (78.3) |  |
| Handgrip strength(kg), Mean ± SD | 28.3 ± 9.3 | 26.3 ± 10.0 | 28.9 ± 9.0 | < 0.001 |
|  | | | | |

**S2 Table. Characteristics of the study participants**
