# Supplementary material for: The relationship between anemia and sleep disturbances among older Chinese adults: The mediating role of handgrip strength
Source: PLoS One. 2025 Oct 9;20(10):e0333673. doi: 10.1371/journal.pone.0333673 (PMC12510644; doi:10.1371/journal.pone.0333673)
Supplement: S3 Table — (DOC) [file pone.0333673.s003.doc]

S3 Table. Associations of anemia and with handgrip strength

| Variables | No | Unadjusted | Model 1 | Model 2 | Model 3 |
| --- | --- | --- | --- | --- | --- |
|  |  | β(95% CI) | β (95% CI) | β(95% CI) | β (95% CI) |
| Anemia | 6057 |  |  |  |  |
| No | 4745 | 1(Ref) | 1(Ref) | 1(Ref) | 1(Ref) |
| Yes | 1312 | -2.95(-3.50~-2.4) | -1.21(-1.61~-0.81) | -1.18(-1.58~-0.78) | -1.20(-1.60~-0.79) |
| Model 1: adjusted for age, gender, educational level, marital status, and residence. Model 2: adjusted for model 1 + smoking status, drinking status, sleep duration, daytime napping duration, and BMI. Model 3: adjusted for model 2 + 14 chronic diseases. Abbreviations: β, Standardized regression coefficients; 95% CI, 95% confidence interval. | | | | | |
