# Supplementary material for: The relationship between anemia and sleep disturbances among older Chinese adults: The mediating role of handgrip strength
Source: PLoS One. 2025 Oct 9;20(10):e0333673. doi: 10.1371/journal.pone.0333673 (PMC12510644; doi:10.1371/journal.pone.0333673)
Supplement: S4 Table — (DOC) [file pone.0333673.s004.doc]

S4 Table. Subgroup analysis of the association between anemia and sleep disturbance

| Subgroup | Total | Event (%) | OR (95%CI) | P for interaction |
| --- | --- | --- | --- | --- |
| Sex |  |  |  | 0.53 |
| Male | 716 | 435 (60.8) | 1.12 (0.93~1.36) |  |
| Female | 596 | 258 (43.3) | 1.23 (1.01~1.51) |  |
| Residence |  |  |  | 0.93 |
| Rural | 886 | 480 (54.2) | 1.18 (0.99~1.4) |  |
| Urban | 426 | 213 (50) | 1.17 (0.92~1.48) |  |
| Marital, Status |  |  |  | 0.55 |
| Married and living with a spouse | 976 | 498 (51) | 1.22 (1.04~1.43) |  |
| Married but living without a spouse | 36 | 19 (52.8) | 0.62 (0.24~1.6) |  |
| Single, divorced, and windowed | 300 | 176 (58.7) | 1.09 (0.8~1.48) |  |
| Education Status |  |  |  | 0.48 |
| Elementary school or below | 1120 | 605 (54) | 1.16 (0.99~1.34) |  |
| Middle school or above | 192 | 88 (45.8) | 1.32 (0.92~1.9) |  |
| Smoking Status |  |  |  | 0.37 |
| Yes | 553 | 242 (43.8) | 1.1 (0.89~1.35) |  |
| No | 759 | 451 (59.4) | 1.25 (1.04~1.51) |  |
| Drinking Status |  |  |  | 0.78 |
| Drink but less than once a month | 101 | 48 (47.5) | 1.00 (0.59~1.69) |  |
| Drink more than once a month | 924 | 509 (55.1) | 1.16 (0.98~1.37) |  |
| Non-drinker | 287 | 136 (47.4) | 1.26 (0.94~1.67) |  |
| BMI group |  |  |  | 0.62 |
| Normal | 165 | 84 (50.9) | 0.99 (0.63~1.55) |  |
| Obesity | 879 | 467 (53.1) | 1.16 (0.97~1.38) |  |
| Overweight | 224 | 122 (54.5) | 1.14 (0.82~1.57) |  |
| Underweight | 32 | 14 (43.8) | 0.72 (0.3~1.74) |  |
| 14 chronic conditions, n (%) |  |  |  | 0.39 |
| 0 | 254 | 97 (38.2) | 0.97 (0.7~1.34) |  |
| 1 | 305 | 140 (45.9) | 1.2 (0.9~1.6) |  |
| ≥2 | 753 | 456 (60.6) | 1.24 (1.03~1.49) |  |
| The reference group was non-anemia. Adjusted for age, sex, educational level, marital status, residence, smoking status, drinking status, BMI, sleep duration, daytime napping duration, and 14 chronic conditions. Abbreviations: OR, odds ratio; 95% CI, 95% confidence interval. | | | | |
