# Supplementary material for: The relationship between anemia and sleep disturbances among older Chinese adults: The mediating role of handgrip strength
Source: PLoS One. 2025 Oct 9;20(10):e0333673. doi: 10.1371/journal.pone.0333673 (PMC12510644; doi:10.1371/journal.pone.0333673)
Supplement: S5 Table — (DOC) [file pone.0333673.s005.doc]

S5 Table. Subgroup analysis of the association between handgrip strength and sleep disturbance

| Subgroup | Total | Event (%) | OR (95%CI) | P for interaction |
| --- | --- | --- | --- | --- |
| Sex |  |  |  | 0.68 |
| Male | 3020 | 1763 (58.4) | 0.98 (0.97~0.99) |  |
| Female | 3037 | 1196 (39.4) | 0.98 (0.97~0.99) |  |
| Residence |  |  |  | 0.95 |
| Rural | 3766 | 1892 (50.2) | 0.98 (0.97~0.99) |  |
| Urban | 2291 | 1067 (46.6) | 0.99 (0.98~1.00) |  |
| Marital, Status |  |  |  | 0.06 |
| Married and living with a spouse | 4726 | 2225 (47.1) | 0.99 (0.98~1.00) |  |
| Married but living without a spouse | 143 | 78 (54.5) | 0.95 (0.9~1.01) |  |
| Single, divorced, and windowed | 1188 | 656 (55.2) | 0.96 (0.94~0.98) |  |
| Education Status |  |  |  | 0.84 |
| Elementary school or below | 4830 | 2454 (50.8) | 0.98 (0.97~0.99) |  |
| Middle school or above | 1227 | 505 (41.2) | 1.00 (0.98~1.02) |  |
| Smoking Status |  |  |  | 0.89 |
| Yes | 2874 | 1202 (41.8) | 0.98 (0.97~0.99) |  |
| No | 3183 | 1757 (55.2) | 0.98 (0.97~1.00) |  |
| Drinking Status |  |  |  | 0.66 |
| Drink but less than once a month | 454 | 214 (47.1) | 0.99 (0.96~1.02) |  |
| Drink more than once a month | 4051 | 2095 (51.7) | 0.98 (0.97~0.99) |  |
| Non-drinker | 1552 | 650 (41.9) | 0.99 (0.97~1) |  |
| BMI group |  |  |  | 0.25 |
| Normal | 447 | 233 (52.1) | 0.98 (0.95~1.02) |  |
| Obesity | 3589 | 1773 (49.4) | 0.99 (0.97~1.00) |  |
| Overweight | 1637 | 768 (46.9) | 0.98 (0.96~0.99) |  |
| Underweight | 301 | 144 (47.8) | 1.01 (0.97~1.05) |  |
| 14 chronic conditions, n (%) |  |  |  | 0.58 |
| 0 | 1152 | 420 (36.5) | 0.97 (0.95~0.99) |  |
| 1 | 1353 | 571 (42.2) | 0.98 (0.96~1.00) |  |
| ≥2 | 3552 | 1968 (55.4) | 0.99 (0.98~1.00) |  |
| Adjusted for age, sex, educational level, marital status, residence, smoking status, drinking status, BMI, sleep duration, daytime napping duration, and 14 chronic conditions. Abbreviations: OR, odds ratio; 95% CI, 95% confidence interval. | | | | |
