# Supplementary figures and images for: The relationship between anemia and sleep disturbances among older Chinese adults: The mediating role of handgrip strength
Source: PLoS One. 2025 Oct 9;20(10):e0333673. doi: 10.1371/journal.pone.0333673 (PMC12510644; doi:10.1371/journal.pone.0333673)

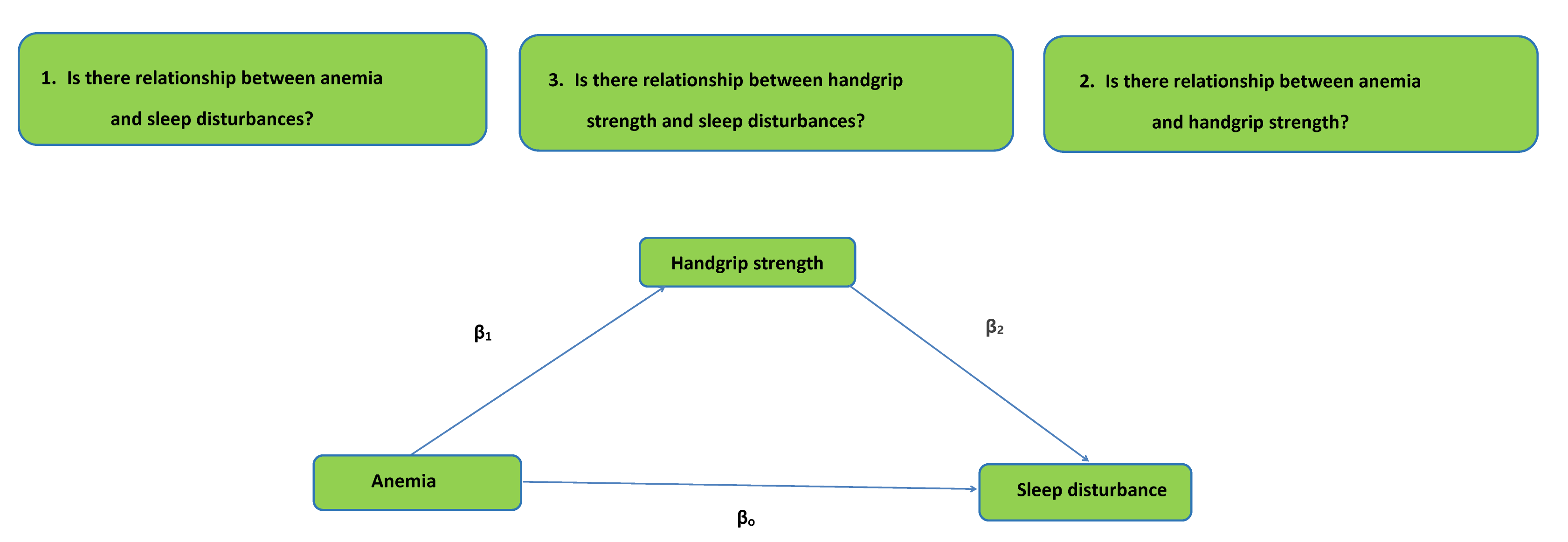

Supplement: S1 Fig — (TIF) [file pone.0333673.s006.tif]

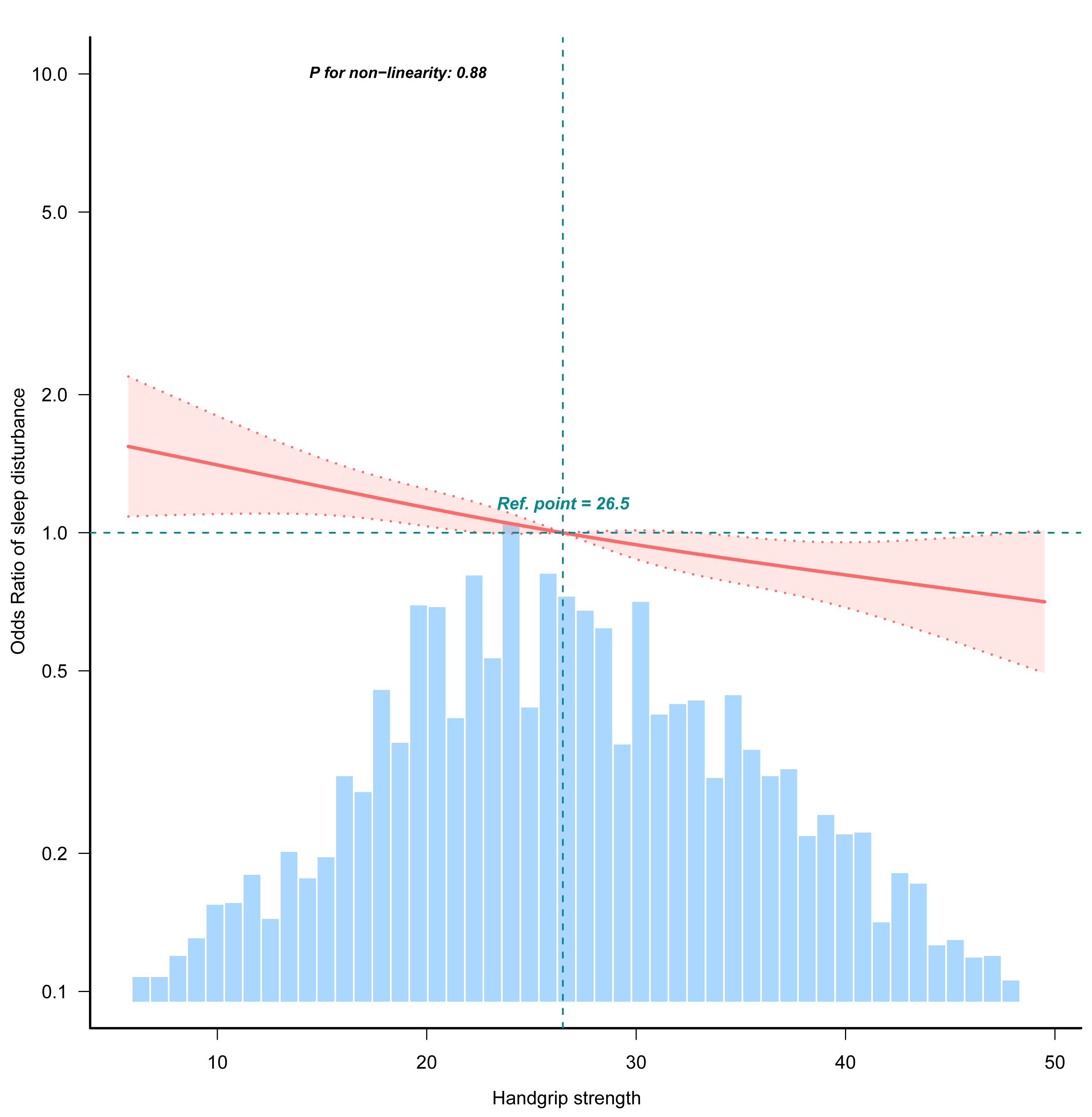

Supplement: S2 Fig — disturbance. The median of the handgrip strength was used as the reference point. Solid and dashed lines represent the predicted value and 95% CI,respectively. Orange bars represent the distribution of the entire cohort. Adjusted for age, sex, educational level, marital status, residence, smoking status, drinking status, BMI, sleep duration, daytime napping duration, and 14 chronic conditions, Only 99% of the data is displayed. (TIF) [file pone.0333673.s007.tif]
