# Supplementary material for: The relationship between anemia and sleep disturbances among older Chinese adults: The mediating role of handgrip strength
Source: PLoS One. 2025 Oct 9;20(10):e0333673. doi: 10.1371/journal.pone.0333673 (PMC12510644; doi:10.1371/journal.pone.0333673)
Supplement: Attachment — Submitted filename: Review_PONE-D-25-13112.pdf [file pone.0333673.s009.pdf]

The relationship between anemia and sleep disturbances among older adults in China: The Mediating Role of handgrip strength

<minor> According to the PLOS ONE submission guidelines, titles should be formatted in sentence case. Please review the guidelines and make the necessary formatting adjustments for the title, headings, references, etc.

Abstract

My comments in the abstract also reflect for the full manuscript, wherever applicable. I may not reiterate these in the full manuscript. I am aware that my comments for the abstract appear longer; however, I hope that these comments are resourceful to increase the quality of your work. Considering the word limit in the abstract, please focus on adding clarity to the most important sections, primarily on methods and the results.

Background

<major>
While you noted that the causal relationship between anemia and sleep disturbances is not fully understood, performing a quick search I found at least one large longitudinal study assessing this relationship (https://doi.org/10.1038/s41598-018-22407-5). Therefore, it is important to critically evaluate and reference such evidence before making broad assertions about causality. Also, as your study is about older adults, I suggest you conduct additional research using keywords associated with older population. Additionally, I don't think talking about causality is necessary here since your study is not longitudinal and does not provide solution to it.

<major>
Is your study specific to only examining the mediating role of handgrip strength, or does it also aim to examine the relationship between anemia and sleep disturbances? If both aspects are included, consider revising your objective to add clarity.

Methods

<minor>
After checking your tables, I found that the number of participants you cited is an analytic sample, but not the original sample size. Kindly add this clarity following the order of operation, as analytic samples are obtained after you follow up with some treatments during your analysis.

<major>
Please check if you have correctly named the dataset you used. Is the correct name China Health and Retirement Longitudinal Study (CHARLS)?

<recommendation>
Please be consistent with the words you choose to define your variable. Since your study is cross-sectional, it might be better to use dependent/independent variables instead of outcome/predictors. Please follow the changes throughout your manuscript.

<major>
Please consider incorporating a clearer explanation of how the major variables in your study are operationalized, specifically the outcome variable, the primary independent variable, and the mediating variable. In its current form, the description hints that sleep disturbance is treated as a numeric variable, which appears inconsistent with the analytic approach you have proposed. Also, please clarify whether you have utilized adjusted or crude multivariable logistic regression for your study.

Results

<recommendation>
The opening statement in the result section is not necessary and redundant. You may delete it.

<recommendation>
Your interpretation is correct; however, I would recommend presenting it using the standard method of defining the odds. Your current interpretation appears as a bivariate assessment, not regression analysis. Also, you don’t need to report p-value after reporting the confidence interval.

<major>
Your abstract has not defined what your adjusted models are. Therefore, please revise and explicitly rewrite the interpretations for the three adjusted models.

Conclusions

<recommendation>
You may avoid using first-person nouns (I/We/Our) and replace it with ‘the study’ or something relevant. In academic setting, this pattern of writing is highly recommended. However, I do not disagree with what you have in your current version.

<major>
Your findings are helpful, but not casual, which can be acknowledged under conclusion. Furthermore, provide a strong recommendation or policy to improve the sleep quality among older adults based on your findings. Merely stating preventing anemia and enhancing handgrip strength do not suffice.

Keywords

<recommendation>
Please arrange your keywords in alphabetical ascending order.

<recommendation>
Your currently listed keywords are only the repetition of what you have in your title. Having keywords other than what you already have in your title increases the search index of your manuscript. I recommend replacing the current keywords with alternate keywords relevant to your study.

<major>
In academic writing, terms like “elderly,” “aged,” and “seniors” are often considered ageist and are best avoided. More inclusive and aging-friendly language is encouraged.

Introduction

<recommendation>
Please be consistent with formatting your headings and subheadings as per PLOS One journals. For example, you have a colon sign after Introduction, but it does not follow the same for the following headings. Please also make sure to change your formatting style to Vancouver.

1st paragraph

Your first paragraph is fairly written. Here are a few of my suggestions:

<major>
Introduce what sleep disturbance is. Integrate if any standard definition exists. You may also need to clarify if sleep disturbance also includes any acute issues such as obstructive sleep apnea, excessive daytime sleepiness, insomnia or any other relevant constructs.
Although you have included some literature discussing the burden of the disease, discussing the burden specific to older adults is important. For example, including a global burden statistics is insightful (https://doi.org/10.1016/j.ssmph.2023.101588), that can follow region-specific prevalence and prevalence in countries comparable to China in terms of settings for older populations.

<minor>
Your assertion that the prevalence of sleep disturbance increases with age requires a citation. Also, integrate somewhere explaining how sleep disturbance differs in older adults (study population) than the overall population?

2nd paragraph

<major> First describe what anemia is. You may also discuss a few points specific to older populations such as how anemia is an important confounder in determining the sleep quality or disturbance in older adults. Are older adults in China more anemic? Are there any contrasting studies?

3rd paragraph

<major> Introduce the concept of handgrip strength in gerontology research.

4th paragraph

This paragraph lacks proper organization and a strong rationale behind conducting your study.

Prior to what you already have, it may be useful to briefly discuss the relationship between the confounders with your independent and dependent variables. Also, there is an increasing tendency to incorporate a theory or a research paradigm to support/test the relevance of your research. I suggest you consider adding a relevant theory that is centralized to your proposed idea, describing how the theory fits well into your research.

Then, provide a strong rationale identifying the existing research gaps and discussing the importance of conducting your research. Following this, provide objective followed by the hypothesis. In your hypothesis, do you mean mediating role of handgrip strength or the moderating role? It is a good idea to provide directions to your proposed hypothesis statement. Also, please consider creating a conceptual framework.

Methods

Data source

<minor>
Is CHARLS a part of longitudinal study or itself a nationally representative longitudinal study?

<major>
Given that the CHARLS dataset is longitudinal, your decision to rely solely on Wave 3 for a cross-sectional analysis warrants a clear and well-reasoned justification—particularly in light of your acknowledgment that research on the causal relationship between anemia and sleep disturbance remains limited. What distinguishes your cross-sectional study from existing ones that have already explored this association? Does your work offer new insights, or does it largely replicate previously established findings? Additionally, it is important to address the limitations inherent in using cross-sectional data to test mediating relationships. Such analyses are generally viewed as exploratory rather than confirmatory, due to the inability to establish temporal ordering and causal direction. Given you already have access to longitudinal data, why motivates you to prefer a cross-sectional analysis? Please, clarify the rationale behind focusing exclusively on a single wave for establishing the validity and contribution of your study.

<major>
The size of the older population (>=60 years) in your study is 9,956. Considering this number, the final analytic sample is 6,057, which accounts for a data loss of 3,899 (39%). Please acknowledge this explicitly to be transparent with your data handling. On the other hand, I don't think multiple imputations on the dependent variable is a good idea. However, if you have other ideas to treat missing values better, you can go with alternate ideas.

<recommendation>
Including a PRISMA flowchart can help readers understand at ease how you reach your final analytic sample size.

Assessment of sleep disturbance, anemia, and handgrip strength

<major>

When you operationalize your outcome variable i.e., sleep disturbance, please be explicit and clear to provide correct terminology and be consistent throughout your manuscript. For example, I don't think sleep disturbance and sleep disorder are the same thing, while the latter one should be clinically diagnosed. Please correct me if this is the other way.
Your rationale of converting the 4-point scale into binary is not satisfactory. Justify why you chose to make your dependent variable binary considering the distribution of your dependent variable as well as with reference to the established literature. It is usually observed that the dependent variable is converted into binary without considering a standard rigor and reason to do so.
Also, explain how the data on sleep disturbance, anemia and handgrip strength were collected? Is your study about sleep quality or sleep disturbance? Or, are these two terms the same idea in your study? Clarify this as well.
Your description of how the data on anemia and handgrip strength are collected is not sufficient. Provide a detailed description. Was any standard protocol followed when measuring handgrip strength? Did you use the standard validated instrument? Which company and what model? Measuring handgrip strength in older population requires special attention. Please, consider elaborating the details.

Covariates

<minor>
Provide categories for sex. You should mention all variables you mention latter such as sleep duration, daytime napping together under additional covariates.

<major>
I was expecting either exercise or physical activity as a potential confounder to control. Also, another important variable might be tea/coffee consumption, if any such usual trend or culture exist in China. Additionally, use of any specific medicine that impacts anemia should have been controlled in the regression analysis. Are these variables available in the dataset? If not, this limitation should be acknowledged under limitations.

Statistical methods

<major>
Did you assess multicollinearity among the predictor variables? If so, what threshold did you use for determining concern (e.g., VIF > 2.5)? Was any variable removed due to multicollinearity issues? Additionally, did you conduct diagnostics for influential outliers, and if any were identified, what methods did you use to address them? What was the value under ROC curve for your predictive models?

Did you also perform a bivariate assessment (Table 1 reflects this)? If yes, please explain the methods applied. Alternatively, you may get rid of bivariate assessment and remove p-values from your Table 1, as you already have conducted unadjusted regression analyses, which are closely similar in terms of methodology. Since you have three different adjusted models, did the models differ with each other? Report on the p-values comparing the models.

I don't have much idea of bootstrap resampling. However, please explore for the rigor, considering that your primary independent and dependent variables are categorical, while your mediating variable is continuous.

Results

<recommendation>
Please make sure that you describe the variables in the same order you have under your methods section, and this is also consistent with all the tables and figures you include.

Characteristics of participants

<recommendation>
Please describe your variable in the sequence. The current version is too haphazard to read and match across Table 1. When you include a table categorizing by your dependent variable, include a few important descriptions of how the distribution looks similar or different across the categories for some of the important independent variables of interest and controls.

Table 1

<minor>
Include percentage for your dependent variable. Also, for simplicity, the variable categories can be yes/no under the variable name as "sleep disturbance?".

Associations between anemia, handgrip strength, and sleep disturbance

<recommendation>
Please consider interpreting your results in terms of increased or decreased odds of experiencing sleep disturbance. You also need to describe what variables you controlled for at least for the first two interpretations. Likelihood and odds are not the same thing. Please use the correct term your analyses have utilized to measure the association.

Correlation between key variables

<major>
Correlation is performed when your both dependent and independent variables are continuous. Please use the correct statistical assessment if you are testing the bivariate association.

The mediating role of handgrip strength in the relationship between anemia and sleep Disturbance

<recommendation>
Revise the interpretation in increased or decreased odds.

<major>
The assertion that "handgrip strength serves as a significant mediator of the relationship between anemia and sleep distribution" implies causal inference. A cross-sectional study can only explore the association but not the causal effects. Please revise your statement accordingly. Consider revising all statements in your manuscript where your interpretation infers establishing a causal effect.

Discussion

<minor>
Your discussion is well-written. My only suggestion is to tighten the language further and avoid implying causal relationships that are not directly supported by your cross-sectional study.

<recommendation>
Provide a subheading as "Limitation and strengths" for the relevant section under discussion. The word "firstly," "secondly" lacks formal tone and should be avoided in academic writing. If necessary, you may write as "first," or "second" and so on.

It seems you only discussed limitations but not the strengths. What is the worth of studying without any strengths? Please discuss strengths as well.

Also, future proposition is too short. You may integrate this section under conclusions. Elaborate on your recommendation for future research. Also, write on the future directions for researchers, social workers, clinicians and policymakers.

References

The references still show inconsistencies with the PLOS ONE journal submission guidelines. Please review them carefully and make the necessary corrections to ensure full compliance.

Ensure that all formatting adhere to the submission guidelines and are consistent throughout the manuscript.
