# Supplementary material for: The relationship between anemia and sleep disturbances among older Chinese adults: The mediating role of handgrip strength
Source: PLoS One. 2025 Oct 9;20(10):e0333673. doi: 10.1371/journal.pone.0333673 (PMC12510644; doi:10.1371/journal.pone.0333673)
Supplement: Attachment — Submitted filename: Response to Reviewers.doc [file pone.0333673.s010.doc]

**Response to Editor**

Dear Dr. Qian Wu

Thank you very much for your letter and the comments about our paper **“The Relationship between anemia and sleep disturbances among Older Adults in China: The Mediating Role of handgrip strength (Manuscript ID: 517084bef51b020d)”**. After carefully studying the reviewers' comments and advice, we have made corresponding changes to the paper. Revised sections are identified with red text in the paper.

There are no conflicts of interest regarding this work. All authors have read the revised manuscript and approved its submission to the **PLOS ONE**. Please do not hesitate to contact us if we can be of any further assistance.

Thank you and best regards.

Yours Sincerely

Xiaojiang Zhao

mail: [zhaoxiaojiang2010@163.com](mailto:zhaoxiaojiang2010@163.com)

**Response to Editor**

**Question 1:** Please ensure that your manuscript meets PLOS ONE's style requirements, including those for file naming. The PLOS ONE style templates can be found at https://journals.plos.org/plosone/s/file?id=wjVg/PLOSOne_formatting_sample_main_body.pdf and <https://journals.plos.org/plosone/s/file?id=ba62/PLOSOne_formatting_sample_title_authors_affiliations.pdf>.

**Answer:** Thank you for your valuable suggestions on the manuscript. We have carefully checked and ensured that the manuscript fully complies with the format requirements of PLOS ONE, including the file naming conventions.

**Question 2:** We note that the grant information you provided in the ‘Funding Information’ and ‘Financial Disclosure’ sections do not match. When you resubmit, please ensure that you provide the correct grant numbers for the awards you received for your study in the ‘Funding Information’ section.

**Answer:** Thank you for your careful review of our manuscript and for bringing this important discrepancy to our attention. We sincerely apologize for the oversight in the funding information consistency between sections. We have now carefully verified our records and made the following corrections to ensure complete alignment: Funding Information section:[Updated grant details with correct grant numbers, e.g., "This work was supported by the Philosophy and Social Sciences Foundation of the Anhui Higher Education Institutions of China (2024AH052821) and Philosophy and Social Sciences Foundation of the Anhui Higher Education Institutions of China (2024AH0052823)" ]( lines 307-309). We appreciate your vigilance in helping us maintain the highest standards of accuracy in our publication. Please let us know if any additional clarifications are needed.

**Question 3:** Thank you for stating the following financial disclosure:

“This work was supported by the Philosophy and Social Sciences Foundation of the Anhui Higher Education Institutions of China (2024AH052821 and 2024AH0052823).“Please state what role the funders took in the study. If the funders had no role, please state: "The funders had no role in study design, data collection and analysis, decision to publish, or preparation of the manuscript."If this statement is not correct you must amend it as needed. Please include this amended Role of Funder statement in your cover letter; we will change the online submission form on your behalf.

**Answer:** Thank you for your comment regarding the funding disclosure. The funders (Philosophy and Social Sciences Foundation of the Anhui Higher Education Institutions of China) had no role in study design, data collection and analysis, decision to publish, or preparation of the manuscript. Please find the following statement included in our cover letter: "The funders had no role in study design, data collection and analysis, decision to publish, or preparation of the manuscript." We kindly request the editorial office to update the online submission form accordingly on our behalf. Please let us know if any additional clarification is needed.

**Question 4:** Please include captions for your Supporting Information files at the end of your manuscript, and update any in-text citations to match accordingly. Please see our Supporting Information guidelines for more information: <http://journals.plos.org/plosone/s/supporting-information.>

**Answer:** Thank you for your valuable feedback. We sincerely appreciate your time and effort in reviewing our manuscript. In response to your comment regarding Supporting Information captions and citations, we have: Added detailed captions for all Supporting Information files at the end of the manuscript, following PLOS ONE's guidelines( lines 290-301).

**Response to Reviewers**

Dear Reviewers.

Thank you very much for your letter and the comments about our paper **“The Relationship between anemia and sleep disturbances among Older Adults in China: The Mediating Role of handgrip strength (Manuscript ID: 517084bef51b020d)”**. After carefully studying the reviewers' comments and advice, we have made corresponding changes to the paper. Revised sections are identified with red text in the paper.

There are no conflicts of interest regarding this work. All authors have read the revised manuscript and approved its submission to the **PLOS ONE**. Please do not hesitate to contact us if we can be of any further assistance.

Thank you and best regards.

Yours Sincerely

Xiaojiang Zhao

mail: zhaoxiaojiang2010@163.com

**Reviewer #1:**

Reviewer Summary and Comments Overall Assessment: This manuscript explores the mediating role of handgrip strength in the association between anemia and sleep disturbance among older Chinese adults, using data from the 2015 CHARLS cohort. The paper is written in clear and fluent English, with a well-structured and logically developed narrative. The research question is timely and of potential public health relevance. However, several conceptual and methodological issues limit the interpretability and robustness of the findings. Below, I provide detailed comments by section.

**Question 1:** Abstract & Introduction: The hypothesis is clearly stated, but the rationale for handgrip strength as a mediator rather than a moderator or independent predictor is insufficiently justified. I recommend briefly outlining the plausible biological mechanisms linking anemia, handgrip strength, and sleep disturbance more clearly in the introduction.

**Answer:** Thank you for your constructive suggestions. Following the your suggestion In the introduction, we have added the description of the biological mechanism that links anemia, handgrip strength, and sleep disturbance( lines 66-71).

**Question 2:** section 2.2 – Assessment of Key Variables: Sleep disturbance is assessed using a single self-reported item ("My sleep was restless"), which limits validity and depth. This should be acknowledged as a limitation. I recommend the authors suggest the use of objective, multidimensional sleep-tracking tools (e.g., actigraphy, wearable devices such as Apple Watch or Oura Ring) in future studies to enhance measurement precision and reduce bias. The definition of anemia follows WHO cutoffs but does not account for age-related changes in hemoglobin, potentially leading to misclassification. This should be addressed or at least mentioned.

**Answer:** Thank you for your valuable suggestion. As a response to "The limitations related to assessment of sleep disturbance and anemia"in response, We have revised the relevant content in the discussion section (Line 276-277).

**Question 3:** Section 3.1 – Descriptive Results: The binary classification of sleep disturbance is simplistic and may obscure meaningful gradations. Consider justifying this dichotomization more clearly or exploring alternative modeling strategies.

**Answer:** Thank you again for your positive comments and valuable suggestions. Based on previous studies(PMID: 39670150, PMID: 31332159, and PMID: 40055626 ), we have added a description of binary classification of sleep disturbances in the methods section( lines 100-104).

**Question 4:** Section 3.2 – Regression Analysis (Table 2): The regression models are statistically sound and robust across adjustments. However, effect sizes are modest (e.g., OR = 1.17 for anemia, OR = 0.98 for handgrip strength). Authors should discuss the clinical significance of these findings. The restricted cubic spline (RCS) curve (Supplementary Figure) appears U-shaped rather than linear. Although the test for non-linearity was non-significant (p = 0.88), the visual pattern should be acknowledged. In particular, lower and higher handgrip strength values both seem to be associated with reduced odds of sleep disturbance, with a peak in odds ratio around 26.5 kg. This contradicts the narrative of a simple inverse relationship and should be interpreted with caution.

**Answer:**

Thank you for your suggestions, they are excellent. As a response to "Regression Analysis (Table 2)"in response, thank you again for your recognition of the research methods and statistical robustness. We fully agree that the discussion of the clinical significance of the effect size is very important. Relevant content has now been added to the discussion section( lines 260-264). As a response to "The restricted cubic spline (RCS) curve (Supplementary Figure1)"in response, In our study, Supplementary Figure 1 uses arestricted cubic spline curve (RCS) to investigate the dose-response relationship between handgrip strength and sleep disturbance, adjusting for confounders according to the logistic regression model 3, which showed a non-linear relationship between handgrip strength and sleep disturbance. However, the reference point 26.5 kg in the graph represents the median handgrip strength (Supplementary Figure 1). To address such misunderstandings, we added "The median of the handgrip strength was used as the reference point" in the legend( lines 512).

**Question 5:** Section 3.3 – Correlations and Mediation Analysis: The rationale for the mediation model (anemia → grip strength → sleep disturbance) remains conceptually weak. The manuscript lacks a physiological explanation of how daytime grip strength causally affects nighttime sleep quality. Mediation requires an explanatory pathway, but this is not well substantiated. For example, muscle inactivity during sleep challenges the premise. Correlations are statistically significant but weak (e.g., r = 0.04). The indirect effect accounts for only 15.67% of the total effect. These limitations should be discussed. Also, two sections are labeled

**Answer:** Thank you for raising these important points. We appreciate the opportunity to clarify and strengthen our rationale. Below is our point-by-point response: As a response to "Explanation of the physiological pathway of mediation model"in response, we acknowledge the need for clearer physiological justification and have expanded our discussion( lines 254-261). As a response to "Correlation and mediating effect"in response, We have added the relevant content in the discussion section (Lines 261-265). As a response to "Labeling error"in response, thank you for carefully reviewing our manuscript and pointing out this numbering error. We have conducted a comprehensive review of the chapter numbers and made the following corrections. Thank you again for helping us improve the standardization of the thesis. If there are any other adjustments that need to be made, please inform us at any time.

**Question 6:** Section 3.4 – Subgroup Analyses (Supplementary Tables 1 & 2): Subgroup analyses do not reveal any significant interactions. Several strata are underpowered, with wide confidence intervals. The authors should clarify whether these analyses were hypothesis-driven or exploratory, and explain their relevance. Supplementary Table 1 (labeled Table 3) shows no significant interaction between anemia and sleep disturbance across subgroups. The observed ORs are modest and variable, with wide confidence intervals in several underpowered strata, limiting interpretability. The same applies to “Supplementary Table 2” (labeled Table 4) regarding subgroup analyses of handgrip strength and sleep disturbance — the manuscript would benefit from clearer table referencing and justification of these additional analyses.

**Answer:**

Thank you for your thoughtful comments regarding our subgroup analyses. We appreciate the opportunity to clarify these important points. Regarding the nature of subgroup analyses, we acknowledge that our presentation could have been clearer. These analyses were primarily exploratory, intended to examine whether the observed associations between anemia/handgrip strength and sleep disturbance might differ across population subgroups. We have refined the relevant content in the Subgroup Analyses section(Lines 184-199). Additionally, regarding the annotation errors in the supplementary table, we have made corrections(S4 Table and S5 Table).

**Question 7:** Discussion: The discussion overstates the strength and causality of findings. Phrases such as "handgrip strength mediates..." should be tempered to "may mediate..." or "suggests a potential mediating role." The narrative includes well-referenced physiological pathways (iron, sarcopenia, sleep-wake regulation), which help contextualize the results. However, this should be clearly separated from what the data actually support. The rationale for mediation remains weak: as muscles are at rest during sleep, the causal pathway from grip strength to sleep remains speculative.

**Answer:** Thank you for your suggestions, we fully agree. Below is our point-by-point response: in response to the expression "the term 'mediates' is inappropriate," we sincerely appreciate the reviewer's insightful comment regarding the need for more cautious language when describing the mediation findings. As suggested, we have revised all definitive causal claims (e.g., 'mediates' → 'may mediate') throughout the Discussion to better reflect the observational nature of our data (Lines 201, 204, 254). As a response to "mediation mechanism"in response, We have added the relevant content in the discussion section (Lines 254-260).

**Question 6:** Limitations & Conclusion: Key limitations are acknowledged, including cross-sectional design, lack of objective sleep measures, and absence of anemia severity stratification. However, the conclusion presents intervention strategies as if causality had been established. This should be revised to reflect the exploratory and correlational nature of the study.

**Answer:** Thank you for your suggestions, we sincerely thank the reviewer for highlighting the need to align our conclusions with the study's correlational design. We have revised the Conclusion section to explicitly emphasize the exploratory nature of our findings and removed any language implying causal recommendations (Line 277-280).

**Question 7:** Recommendation: Major Revision. The study is relevant and methodologically competent, but the theoretical justification for the mediation model needs significant improvement. Claims of mediation and causal direction should be rephrased cautiously, and several methodological limitations should be better addressed. With these revisions, the manuscript could make a valuable contribution to the literature.

**Answer:** Thank you again for your valuable opinions on this article. The insufficiency of the theoretical framework and the problem of causal inference you pointed out are indeed crucial to the quality of the article. We have made the modifications as per your suggestions (see the modification section above).

**Reviewer #2:**

The present study investigates the relationship between anemia and sleep disturbances among older adults in China: The mediating role of handgrip strength. The present study investigates the mediating role of handgrip strength in the relationship between anemia and sleep disturbances. However, some comments have been found while reading the paper which need to be revised.The following comments are described as given:

**Question 1:** The quantitative results beside the qualitative results should be added in the abstract should.

**Answer:**

We sincerely appreciate your constructive suggestion. According to the suggestions, we have added the key quantitative findings in the abstract(Lines 28-30).

**Question 2:** The structure of the paper should be clearly described at the end of introduction section.

**Answer:** We sincerely appreciate your constructive suggestion. As recommended, we have added a clear outline of the paper’s structure at the end of the introduction section (Lines 75-78), to enhance readability and logical flow.

**Question 3:** Literature review should be separate from the introduction since the novelty is not clear.

**Answer:** We sincerely appreciate your constructive suggestion. According to the suggestions, we have adjusted the introduction section to clarify the novelty (Lines 66-71, 75-78).

**Question 4:** What is the research gap between the present study and studies in the literature review? Authors should highlight this importance at the end of literature review. What is the novelty of the present work in comparison with other works?

**Answer:**

We appreciate your insightful suggestions and are in full agreement with them. Below, we provide a detailed point-by-point response: Regarding the inquiry, "What is the research gap between the present study and studies in the literature review?" we have incorporated pertinent content into the introduction section (Lines 72-75) As advised. Similarly, in response to the question "What is the novelty of the present work in comparison with other works?" we have also added the relevant content to the introduction section (Lines 75-78).

**Question 5:** In order to extend the domain of the present study in comparison with other studies and increase the contribution of the present paper with other evaluation methods in anemia and sleep disturbances among older adults and other fields, the following references could be added which are: • Orexin Neurons to Sublaterodorsal Tegmental Nucleus Pathway Prevents Sleep Onset REM Sleep-Like Behavior by Relieving the REM Sleep Pressure. Research, 7, 0355. doi: 10.34133/research. 0355 • The relationship between handgrip strength and cognitive function among older adults in China: Functional limitation plays a mediating role. Journal of Affective Disorders, 347, 144-149. • Nip it in the bud: the impact of China’s large-scale free physical examination program on health care expenditures for elderly people. Humanities and Social Sciences Communications, 12(1), 27. doi: 10.1057/s41599-024-04295-5 • Factors associated with low handgrip strength in older people: data of the Study of Chronic Diseases (Edoc-I). BMC Public Health, 20, 1-10.

**Answer:** We sincerely appreciate the reviewer's thoughtful comments and valuable suggestions for improving our manuscript. As suggested, we have now incorporated the following key references to broaden the discussion and strengthen the interdisciplinary connections of our study.

**Question 6:** Authors should add a section regarding the data description, initial data statistics, sample stability testing. How did authors understand that the sample data is enough? Has the survey response stability been implemented? Please add a section regarding this importance.

**Answer:** Thank you for your valuable comments on our thesis. We fully agree on the importance of data description, statistical analysis and sample stability testing for the rigor of the research. According to your suggestion, we have added new content in the revised manuscript(Supplementary Table 1, Lines 169-170).

**Question 7:** In the research method, what statistical tests have been conducted to evaluate the normality or abnormality of data? Wasn’t it better to use some statistical tests for evaluating normality or abnormality of data and then use Pearson or Spearman based on the data for investigating the correlation?

**Answer:** We appreciate the reviewer’s insightful comment. Below, we provide a detailed point-by-point response: in response to the question " regarding data normality assessment?" We used the Shapiro-Wilk test to evaluate normality, indicating that grip strength was normally distributed (p < 0.05). we have also added the relevant content to the Statistical methods section(Lines 140-146). In response to the question " Correlation statistical method?" In this study, the independent effects of anemia and handgrip strength on sleep disturbances were evaluated by gradually adjusting multiple covariates. Meanwhile, sleep disturbances is binary and multiple factors need to be analyzed. Therefore, multivariable logistic regression analysis was selected for this study.

**Question 8:** Why multivariable logistic regression analysis was used? How about simple regression or multi regression models? Why authors didn’t use the mentioned models? How did they decide to use multivariable logistic regression analysis?

**Answer:** Thank you for your thoughtful question regarding our choice of statistical methods. We appreciate the opportunity to clarify our analytical approach. Multiple Regression Models and Multivariable Logistic Regression Analysis are two different statistical methods, mainly used for different data types and analysis purposes. If the dependent variable is continuous, use Multiple Regression. If the dependent variable is categorical (especially binary), use Multivariable Logistic Regression. Since the dependent variable of this study is a binary categorical variable, Multivariable Logistic Regression Analysis is used.

**Question 9:** Conclusion part should be extended based on the whole paper since it is the main part of the present work.

**Answer:** We sincerely appreciate your constructive feedback. As suggested, we have expanded the Conclusion section, ensuring alignment with the overall paper(Lines 292-293).

**Reviewer #3:**

This study offers a valuable contribution by examining the relationship between anemia and sleep disturbances among older adults in China, particularly relevant in the current context where many countries are transitioning into aging societies. The findings are of particular importance given the implications of poor sleep quality for overall well-being and life satisfaction among the elderly. Notably, the study also explores the mediating role of handgrip strength, a novel angle that may help to inform strategies aimed at improving sleep quality. However, I would like to point out several areas where further development and refinement are warranted General

**Question 1:** Comments • Manuscript structure: The writing throughout the manuscript would benefit from refinement for clarity and academic tone. Redundant expressions (e.g., "association relationship") should be revised for conciseness and precision.

**Answer:** We sincerely appreciate your valuable feedback regarding the clarity and academic tone of our manuscript. We have carefully revised the text to improve precision and eliminate redundant expressions throughout the paper (Lines 12, 23, 25).

**Question 2:** • References: The citation style needs to be standardized according to journal guidelines. Additionally, the full text or supplemental material should be provided for complete data transparency.

**Answer:** We sincerely appreciate the reviewer’s valuable feedback. We have carefully addressed the concerns regarding citation standardization and data transparency as follows: As a response to "Citation Style Standardization", in response, We have reformatted all references in the manuscript to strictly adhere to the journal’s guidelines. As a response to "Full Text"in response, We have now uploaded the complete "the raw data" (see "the raw data").

**Question 3:** Section-Specific Comments 1.

Abstract • The objective section could be more clearly articulated, and redundant phrasing should be avoided. For example, the sentences: “This study seeks to examine the mediating role of handgrip strength in the relationship between anemia and sleep disturbances. ” and“Additionally, bootstrap tests were performed to assess the mediating role of handgrip strength in the association between anemia and sleep disturbances. ” essentially convey the same point and should be consolidated. • In the results section, consider replacing the word "correlation" if the results are based on odds ratios (OR), which imply associative or causal inference rather than simple correlation.

**Answer:** We sincerely appreciate your constructive comments. Please find our revisions below: We have consolidated the redundant statements about the mediation analysis and refined the objective description (Lines 75-78) The term "correlation" has been replaced with "association" to better reflect the analytical approach (Lines 25, 27-30). We hope these revisions address your concerns effectively.

**Question 4:** Introduction • The citation to Zhong et al., 2024, which states a relationship between grip strength and sleep disturbances, appears to be inaccurate. Based on the referenced study, Zhong et al. examined waist circumference and sleep disorders—not grip strength. This needs to be corrected.

• The rationale for proposing handgrip strength as a mediator is currently weak. A theoretical or empirical basis should be introduced to support its mediating role—such as references to prior studies or physiological mechanisms that connect muscle strength, anemia, and sleep disturbances.

• The statement “several studies” is misleading when only one source (e.g., Denny et al., 2006) is cited.Multiple citations (≥2) are expected to support such a claim.

• Vague terms like "complexity" should be elaborated upon or supported with concrete examples or sources—what constitutes the "complexity" of sleep disturbances?

**Answer:** Thank you for your suggestions, we fully agree. Below is our point-by-point response: in response to the expression "Regarding the citation error of Zhong et al., 2024' is inappropriate," we sincerely appreciate the reviewer’s careful reading. The citation to Zhong et al. was indeed incorrect, as the study focused on waist circumference rather than grip strength. We have removed this reference and replaced it with relevant studies explicitly linking handgrip strength to sleep disturbances (Lines 60-66). As a response to "mediation mechanism"in response, We agree with the reviewer that further justification for the mediating role of handgrip strength is needed. In the revised manuscript, we have expanded the introduction (Line66-71). As a response to "several studies"in response, We apologize for the oversight. The phrase “several studies” has been revised to specify the exact number of supporting references. For example, in addition to Denny et al. (2006), We have added new literatures(Line 49, 50).

**Question 5:** Methods

• The rationale behind using the CHARLS questionnaire to assess sleep disturbances should be justified. Does this single-question format reliably capture sleep quality among older adults? Consider comparing it with validated tools like the Pittsburgh Sleep Quality Index (PSQI).

• Details on how handgrip strength was measured are limited. Was the method aligned with WHO, NIH, or other standardized protocols? Including a reference or illustration of the assessment method would enhance clarity.

**Answer:**

We appreciate the reviewer’s insightful comment. we provide a detailed point-by-point response: in response to the question " regarding the measurement of sleep disturbances". We acknowledge the reviewer’s valid concern regarding the measurement of sleep disturbances. The CHARLS questionnaire’s single-item sleep assessment was selected primarily due to its alignment with the study’s large-scale, population-based design, which prioritized feasibility and consistency across a nationally representative sample. We recognize your effective concern about the measurement of sleep disturbances.

In response to the question "how handgrip strength was measured are limited". We thank you for highlighting this gap. Grip strength was measured using a standardized protocol consistent with the NIH toolkit and WHO recommendations: Participants conducted two tests on each hand using a dynamometer, with their elbows sitting at a 90° Angle, and recorded the highest value during the test. Our method section has been modified(Lines 113-120).

**Question 5:** Results

Table 1: While p-values are presented, the specific statistical tests used (e.g., chi-square, t-test) are not indicated. In addition, the standard deviations for handgrip strength vary considerably between groups. Reporting an effect size (e.g., Cohen’s d) would enhance interpretability.

• Table 2: The odds ratio for handgrip strength should specify the unit, such as “per 1 kg increase” or “per standard deviation increase,” to clarify interpretation.

• Table 3: Although correlations may be statistically significant, the magnitude is very weak and should be acknowledged as such.

• Figure 2 should be revised to include additional methodological details, such as the statistical technique used (e.g., bootstrapping), and the confidence intervals (CIs) for the indirect effect, if available. Including this information would enhance the completeness and interpretability of the mediation analysis.

**Answer:** We sincerely appreciate the reviewer’s thoughtful feedback and constructive suggestions, which have significantly improved the clarity and rigor of our manuscript. Below, we address each point in detail: in response to the question "Table 1: Statistical Tests and Effect Sizes". We have now specified the statistical tests used (Independent-sample t tests for continuous variables and chi-square tests for categorical variables were used) in the table footnote(Table 1).We strongly agree with your point of view. Combined with previous studies, our research only calculated the *p*-values. We will consider your suggestion in future studies. Please do not hesitate to get in touch in case of further questions. In response to the question "Table 3: Magnitude of Correlations". We agree that while correlations are statistically significant, their weak magnitude (e.g., r = 0.04) limits clinical interpretability. We now emphasize this in the Discussion Section(Lines 261-265). In response to the question "Figure 2: Mediation Analysis Details". We have refined the description of mediation analysis in the methods section and added confidence interval (95% ci) of the indirect effect in Figure 2.

**Reviewer #4:**

Thank you for this important work. I enjoyed reviewing your work. The major strength of this manuscript were the flow your writing and the methodology aspects including a longitudinal dataset. However, I have some critical points for your to address in the attached document. If you find some of my comments not relevant, please feel free to respond accordingly. I wish you and your coauthors the best, and I hope that my comments will help enhance the quality of your work.

**<minor>** According to the PLOS ONE submission guidelines, titles should be formatted in sentence case. Please review the guidelines and make the necessary formatting adjustments for the title, headings, references, etc.

**Answer:** We sincerely thank you for your constructive suggestions. According to the suggestions and referring to the PLOS ONE submission guidelines, we have made the necessary format adjustments to the title, headings, references, etc.

**Abstract**

**Background**

**<major>**

While you noted that the causal relationship between anemia and sleep disturbances is not fully understood, performing a quick search I found at least one large longitudinal study assessing this relationship (<https://doi.org/10.1038/s41598-018-22407-5>). Therefore, it is important to critically evaluate and reference such evidence before making broad assertions about causality. Also, as your study is about older adults, I suggest you conduct additional research using keywords associated with older population. Additionally, I don’t think talking about causality is necessary here since your study is not longitudinal and does not provide solution to it.

**Answer:** We sincerely thank you for your insightful comments and for drawing our attention to this important research. We agree that it is crucial to critically evaluate the existing evidence before discussing the relevant relationships. According to your suggestion, we focused on describing the elderly. Also, as you pointed out, our cross-sectional design does not allow causal inference. We have revised the expression now(Lines 11-15).

**<major>**

Is your study specific to only examining the mediating role of handgrip strength, or does it also aim to examine the relationship between anemia and sleep disturbances? If both aspects are included, consider revising your objective to add clarity.

**Answer:** We sincerely thank the reviewers for raising this valuable question. As is correctly pointed out, our study is the anemia and sleep disturbances association and the mediating role of handgrip strength in this relationship. To address the issue you are concerned about, we have made some modifications to this part(Lines 11-15).

**Methods**

**<minor>**

After checking your tables,I found that the number of participants you cited is an analytic sample, but not the original sample size. Kindly add this clarity following the order of operation, as analytic samples are obtained after you followup with some treatments during your analysis.

**Answer:** Thank you for your detailed review of the description of the sample size. The suggestions you put forward are very pertinent. We have made revisions in the revised manuscript**(Lines 16-18)**.

**<major>**

Please check if you have correctly named the dataset you used. Is the correct name China Health and Retirement Longitudinal Study (CHARLS)?**Answer:** Thank you for your correction. We have corrected the name of the dataset to 'China Health and Retirement Longitudinal Study (CHARLS)' as required(Lines 16, 17).

**<recommendation>**

Please be consistent with the words you choose to define your variable. Since your study is cross-sectional, it might be better to use dependent/independent variables instead of outcome/predictors. Please follow the changes throughout your manuscript.

**Answer:** Thank you for your thoughtful advice. We agree that maintaining consistent terms is very important for clarity. We have revised the manuscript and used "dependent variable/independent variable" throughout the text because this is more in line with the cross-sectional nature of our research(Lines 18).

**<major>**

Please consider incorporating a clearer explanation of how the major variables in your study are operationalized, specifically the outcome variable, the primary independent variable, and the mediating variable. In its current form, the description hints that sleep disturbance is treated as a numeric variable, which appears inconsistent with the analytic approach you have proposed. Also, please clarify whether you have utilized adjusted or crude multivariable.

**Answer:** Thank you for your insightful comments regarding the operationalization of variables and regression methodology. As a The response to "primary independent variable" in the response. Thank you again for your positive comments and valuable suggestions. We changed the relevant description(Lines 18, 19). As a The response to "Regression Analysis Clarification" in the response. We made the relevant description(Lines 21).

**Results**

**<recommendation>**

The opening statement in the result section is not necessary and redundant. You may delete it.

**Answer:** We thank the reviewers for pointing this out. You're right. This opening statement is indeed redundant. We have deleted it as suggested and made adjustments to the relevant parts.

**<recommendation>**

Your interpretation is correct; however, I would recommend presenting it using the standard method of defining the odds. Your current interpretation appears as abivariate assessment,not regression analysis. Also, you don’t need to report p-value after reporting the confidence interval.

**Answer:** We express our gratitude for the guidance of the statistical report. According to your suggestion, we deleted the redundant p-values of the already reported confidence intervals and focused on the effect size and interval estimation(Lines 26, 27).

**<major>**

Your abstract has not defined what your adjusted models are. Therefore, please revise and explicitly rewrite the interpretations for the three adjusted models.

**Answer:** We sincerely thank you for your constructive feedback. We have described what the adjusted models are, but due to word count limitations, we have detailed three models in the Statistical methods section to enhance transparency(Lines 149-153). Please do not hesitate to get in touch in case of further questions.

**Conclusions**

**<recommendation>**

You may avoid using first-person nouns (I/We/Our) and replace it with ‘the study ’ or something relevant. In academic setting, this pattern of writing is highly recommended. However, I do not disagree with what you have in your current version.

**Answer:** Thank you for the reviewers' suggestions. We have replaced the first-person pronouns (such as I/We/Our) with objective expressions like 'the study' as per the suggestions(Lines 31, 94, 126, etc).

**<major>**

Your findings are helpful, but not casual, which can be acknowledged under conclusion. Furthermore, provide a strong recommendation or policy to improve the sleep quality among older adults based on your findings. Merely stating preventing anemia and enhancing handgrip strength do not suffice.

**Answer:** Thank you for your recognition of the research value and the valuable suggestions you have put forward. We fully agree with your point of view. We have supplemented the relevant content in the revised manuscript(Lines 33,34).

**Keywords**

**<recommendation>**

Please arrange your keywords in alphabetical ascending order.

**Answer:** We sincerely thank you for your precious time and constructive suggestions. We arrange the key words in ascending alphabetical order(Lines 35).

**<recommendation>**

Your currently listed keywords are only the repetition of what you have in your title. Having keywords other than what you already have in your title increases the search index of your

manuscript. I recommend replacing the current keywords with alternate keywords relevant to your study.

**Answer:** We sincerely thank you for your constructive suggestions on optimizing keywords to enhance the discoverability of the manuscripts. We have added new keywords(Lines 35).

**<major>**

In academic writing, terms like “elderly,” “aged,” and “seniors” are often considered ageist and are best avoided. More inclusive and aging-friendly language is encouraged.

**Answer:** We sincerely thank the reviewers for their thoughtful comments on the importance of inclusive language for the elderly. We fully agree that avoiding the use of age-discriminatory terms is in line with contemporary academic standards and promotes respect. Just as suggested, we have replaced it with a more neutral alternative word(Lines 14, 18, 32, etc).

**Introduction**

**1st paragraph**

Your first paragraph is fairly written. Here are a few of my suggestions:

**<major>**

Introduce what sleep disturbance is. Integrate if any standard definition exists. You may also need to clarify if sleep disturbance also includes any acute issues such as obstructive sleep apnea, excessive daytime sleepiness, insomnia or any other relevant constructs. Although you have included some literature discussing the burden of the disease, discussing the burden specific to older adults is important. For example, including a global burden statistics is insightful [(https://doi.org/10.1016/j.ssmph.2023.101588)](https://doi.org/10.1016/j.ssmph.2023.101588), that can follow region- specific prevalence and prevalence in countries comparable to China in terms of settings for older populations.

**Answer:** Thank you for your insightful suggestions. We agree that emphasizing the disease burden specific to the elderly, especially in the context of global and regional comparability, will strengthen our manuscript. In response to your comments, we are now: increases the reference study (10.1016 / j.s SMPH) 2023.101588 the global burden of statistical data, the key data highlighted the aging of the population(Lines 42, 43).

**<minor>**

Your assertion that the prevalence of sleep disturbance increases with age requires a citation. Also, integrate somewhere explaining how sleep disturbance differs in older adults (study

population) than the overall population?

**Answer:** We sincerely thank the reviewers for their valuable comments. We have now added some citations to support the age-related prevalence of sleep disturbance (Lines 40-42).

**2nd paragraph**

**<major>** First describe what anemia is. You may also discuss a few points specific to older populations such ashow anemia is an important confounder in determining the sleep quality or disturbance in older adults. Are older adults in China more anemic? Are there any contrasting studies?

**Answer:** We appreciate your valuable suggestions, which have given us the opportunity to clarify this important concept. We have added the description of the concept of anemia **(Lines 47, 48**).

**<major>** Introduce the concept of handgrip strength in gerontology research.

**Answer:** Thank you for your valuable suggestions on the role of grip strength in gerontology research. We have added relevant content(Lines 58).

**4th paragraph**

This paragraph lacks proper organization and a strong rationale behind conducting your study. Prior to what you already have, it maybe useful to briefly discuss the relationship between the confounders with your independent and dependent variables. Also, there is an increasing tendency to incorporate a theory or a research paradigm to support/test the relevance of your research. I suggest you consider adding a relevant theory that is centralized to your proposed idea, describing how the theory fits well into your research.

Then, provide a strong rationale identifying the existing research gaps and discussing the importance of conducting your research. Following this, provide objective followed by the hypothesis. In your hypothesis, do you mean mediating role of handgrip strength or the

moderating role? It is a good idea to provide directions to your proposed hypothesis statement. Also, please consider creating a conceptual framework.

**Answer:** We sincerely thank the reviewers for their constructive feedback. We have rewritten this section, which helps us strengthen the theoretical foundation and clarity of the paper(Lines 75-78).

**Methods**

**Data source**

**<minor>**

Is CHARLS a part of longitudinal study or itself a nationally representative longitudinal study?

**Answer:** CHARLS itself is a longitudinal study with national representativeness.

**<major>**

Given that the CHARLS dataset is longitudinal, your decision to rely solely on Wave 3 for a cross-sectional analysis warrants a clear and well-reasoned justification—particularly in light of your acknowledgment that research on the causal relationship between anemia and sleep disturbance remains limited. What distinguishes your cross-sectional study from existing ones that have already explored this association? Does your work offer new insights, or does it largely replicate previously established findings? Additionally, it is important to address the limitations inherent in using cross-sectional data to test mediating relationships. Such analyses are generally viewed as exploratory rather than confirmatory, due to the inability to establish temporal ordering and causal direction. Given you already have access to longitudinal data, why motivates you to prefer a cross-sectional analysis? Please, clarify the rationale behind focusing exclusively on a single wave for establishing the validity and contribution of your study.

**Answer:** Thank you for the important questions you raised about our research methods. Regarding our decision to use CHARLS Wave 3 data for cross-sectional analysis rather than longitudinal analysis, we understand your concerns and are willing to explain our considerations in detail. First of all, our research builds on previous literature on the association between anemia and sleep disturbances, but we focused on older Chinese adults and explored the mediating role of handgrip strength in the association between anemia and sleep disturbances. Secondly, the response rate and data quality of Wave 3 are the most ideal among all waves of CHARLS. In the future, we will follow your suggestion and conduct research using the complete longitudinal data of CHARLS.

**<major>**

The size of the older population (>=60 years) in your study is 9,956. Considering this number, the final analytic sample is 6,057, which accounts for a data loss of 3,899 (39%). Please acknowledge this explicitly to be transparent with your data handling. On the other hand, I don’t think multiple imputations on the dependent variable is a good idea. However, if you have other ideas to treat missing values better, you can go with alternate ideas.

**Answer:** We sincerely thank you for emphasizing this important issue. As mentioned earlier, 39% (3,899/9,956) of eligible participants were excluded due to the lack of data. We now clearly discuss this limitation of the study in the Discussion Section(Lines 306-308).

**<major>**

When you operationalize your outcome variable i.e., sleep disturbance, please be explicit and clear to provide correct terminology and be consistent throughout your manuscript. For example, I don’t think sleep disturbance and sleep disorder are the same thing, while the latter one should be clinically diagnosed. Please correct me if this is the other way. Your rationale of converting the 4-point scale into binary is not satisfactory. Justify why you chose to make your dependent variable binary considering the distribution of your dependent variable as well as with reference to the established literature. It is usually observed that the dependent variable is converted into binary without considering a standard rigor and reason to do so. Also, explain how the data on sleep disturbance, anemia and handgrip strength were collected? Is your study about sleep quality or sleep disturbance? Or, are these two terms the same idea in your study? Clarify this as well. Your description of how the data on anemia and handgrip strength are collected is not sufficient. Provide a detailed description. Was any standard protocol followed when measuring handgrip strength? Did you use the standard validated instrument? Which company and what model? Measuring handgrip strength in older population requires special attention. Please, consider elaborating the details.

**Answer:** Thank you for your suggestions, they are excellent. As a response to "sleep disturbance"in response, we thank you for pointing out the important difference between sleep disturbance and sleep disorder. Indeed, as you said, sleep disorder is a medical condition that requires clinical diagnosis. In our study, what we measured was self-reported sleep disturbance. We have uniformly used the term "sleep disturbance" throughout the manuscript. As a response to "Converting the 4-point scale into binary"in response. Thank you again for your positive comments and valuable suggestions. Based on previous studies, We have added a description of binary classification of sleep disturbances in the methods section (lines 123-127). As a The response to "A description of collecting data on anemia and grip strength" in the response. Thank you again for your positive comments and valuable suggestions. We elaborated on the details (lines 105-120).

**Covariates**

**<minor>**

Provide categories for sex. You should mention all variables you mention latter such as sleep duration, daytime napping together under additional covariates.

**Answer:** Thank you for your valuable suggestions on gender classification and covariate handling in our research. In response to the problems you pointed out, we have revised and improved the manuscript(lines 155).

**<major>**

I was expecting either exercise or physical activity as a potential confounder to control. Also, another important variable might be tea/coffee consumption, if any such usual trend or

culture exist in China. Additionally, use of any specific medicine that impacts anemia should have been controlled in the regression analysis. Are these variables available in the dataset? If not, this limitation should be acknowledged under limitations.

**Answer:** Thank you for your detailed review of the research and the valuable suggestions you have put forward. We did not find the confounding factors you mentioned in the dataset. We have acknowledged the deficiencies in the limitations. Thank you again for helping to enhance the rigor of this research. If there are any other suggestions, we are more than willing to make further revisions(lines 316, 317).

**Statistical methods**

**<major>**

Did you assess multicollinearity among the predictor variables? If so, what threshold did you use for determining concern (e.g., VIF > 2.5)? Was any variable removed due to multicollinearity issues? Additionally, did you conduct diagnostics for influential outliers, and if any were identified, what methods did you use to address them? What was the value under ROC curve for your predictive models?

Did you also perform abivariate assessment (Table 1 reflects this)? If yes, please explain the methods applied. Alternatively, you may get rid of bivariate assessment and remove p-values from your Table 1, as you already have conducted unadjusted regression analyses, which are closely similar in terms of methodology. Since you have three different adjusted models, did the models differ with each other? Report on the p-values comparing the models.

I don’t have much idea of bootstrap resampling. However, please explore for the rigor,

considering that your primary independent and dependent variables are categorical, while your mediating variable is continuous.

**Answer:** Thank you for your attention to the issue of Statistical methods. Below is our point-by-point response: As a response to "Regarding multicollinearity Diagnosis "in response, we evaluated the multicollinearity problem among the predictor variables, as detailed in Supplementary Table 2 and(Lines 147). As a response to "Regarding the diagnosis of outliers "in response, our cross-sectional study did not conduct the diagnosis of outliers. Instead, we conducted a Logistic regression model. We are actively exploring these issues, and plan to publish results from these studies in the near future. As a response to "Regarding bivariate analysis and Table 2 ",in response, we understand your suggestions on bivariate analysis. Indeed, the unadjusted regression analysis is similar in method to the bivariate test. By removing the p-values from Table 2, it is indicated in the method section that we mainly rely on the regression analysis rather than the bivariate test(Table 2).

As a response to "Bootstrap Resampling with Categorical and Continuous Variables",in response. Thank you for your insightful comment regarding the use of bootstrap resampling in our analysis. We appreciate the opportunity to clarify our methodological approach.We employed bootstrap resampling (e.g., 1.000 iterations) to address potential non-normality in the sampling distribution of the mediation effect, particularly given the categorical nature of our independent (X) and dependent (Y) variables. For the categorical anemia (k levels) and sleep disturbance, we used dummy coding (k-1 dummy variables) to ensure compatibility with the continuous mediator model. Bootstrap resamples were drawn stratified by anemia levels to maintain the original group proportions, preserving the study design's integrity. The mediation model was estimated using a generalized structural equation modeling (GSEM) framework, with:A linear regression path (anemia→ sleep disturbance). A logistic/probit regression path (handgrip strength →sleep disturbance, accounting for sleep disturbance's categorical nature). Bootstrap CIs for the indirect effect (anemia → handgrip strength → sleep disturbance) were derived from the product of coefficients (a×b), with bias-corrected adjustments to improve accuracy in small samples.

**Results**

**<recommendation>**

Please make sure that you describe the variables in the same order you have under your methods section, and this is also consistent with all the tables and figures you include.

**Answer: T**hank you for your careful review and constructive comment. We sincerely apologize for the inconsistency in the order of variable descriptions across the Methods, Results, Tables, and Figures.We have now thoroughly revised the manuscript to ensure that: 1.All variables are described in the same order as they appear in the Methods section. 2. The sequence in Tables and Figures has been adjusted to align with this order. The changes have been implemented in the revised manuscript (Lines 186,214-220). Please let us know if further adjustments are needed. Thank you again for your valuable feedback.

**<recommendation>**

Please describe your variable in the sequence. The current version is too haphazard to read and match across Table 1. When you include a table categorizing by your dependent variable, include a few important descriptions of how the distribution looks similar or different across the categories for some of the important independent variables of interest and controls.

**Answer:** Thank you for your valuable suggestions on the description of variables and the presentation of tables in the paper. We fully agree with you that the current variable description does indeed require a more systematic organization. We have made improvements(Lines193-201).

**Table 1**

**<minor>**

Include percentage for your dependent variable. Also, for simplicity, the variable categories can be yes/no under the variable name as “sleep disturbance?” .

**Answer:** We appreciate your constructive suggestions. We have now revised Table 1 to: (1) Include percentage values for the dependent variable ("Sleep disturbance?") alongside absolute counts, and (2) Simplify the categories to "Yes/No" under the variable name for clarity.The updated table better aligns with the journal's readability standards, and we believe these changes enhance the interpretability of our results(Table 1).

**Associations between anemia, handgrip strength, and sleep disturbance**

**<recommendation>**

Please consider interpreting your results in terms of increased or decreased odds of experiencing sleep disturbance. You also need to describe what variables you controlled for at least for the first two interpretations. Likelihood and odds are not the same thing. Please use the correct term your analyses have utilized to measure the association.

**Answer:** Thank you for your valuable comments on the statistical interpretation in our research. We have carefully considered your suggestion and made corresponding revisions (Lines204-206). We believe that these adjustments have made our statistical expressions more accurate and professional. Thank you again for your detailed review and constructive suggestions.

**Correlation between key variables**

**<major>**

Correlation is performed when your both dependent and independent variables are continuous. Please use the correct statistical assessment if you are testing the bivariate association.

**Answer:** We appreciate your identification of this issue. Upon further review, we recognized that "anemia" is a categorical variable, whereas "handgrip strength" is a continuous variable. Therefore, we have changed the Correlation analysis method between anemia and handgrip strength to Linear regression analysis. Additionally, we have omitted the section titled "Correlation between key variables" from the results. The findings regarding the association between anemia and handgrip strength have been incorporated into the section "Associations between anemia, handgrip strength, and sleep disturbance."(Lines209-211).

**The mediating role of handgrip strength in the relationship between anemia and sleep Disturbance**

**<recommendation>**

Revise the interpretation in increased or decreased odds.

**Answer:** We thank the reviewers for their comments on clarifying the explanation of the odds ratio. **According to the suggestions, we have made corresponding modifications.**

**<major>**

The assertion that “handgrip strength serves as a significant mediator of the relationship between anemia and sleep distribution” implies causal inference. Across-sectional study can only explore the association but not the causal effects. Please revise your statement accordingly. Consider revising all statements in your manuscript where your interpretation infers establishing a causal effect.

Answer: We sincerely thank the reviewers for their insightful comments on the interpretation of the mediation analysis in our cross-sectional study. In response to this concern, we revised all the statements in the manuscript that might imply causal relationships to clarify that our findings only reflect associations consistent with potential mediating effects(Lines228-230).

**Discussion**

**<minor>**

Your discussion is well-written. My only suggestion is to tighten the language further and avoid implying causal relationships that are not directly supported by your cross-sectional study.

**Answer:** Thank you for your constructive suggestions. We have changed the language, to avoid implied

**<recommendation>**

Provide a subheading as “Limitation and strengths” for the relevant section under discussion. The word “firstly,” “secondly” lacks formal tone and should be avoided in academic writing. If necessary, you may write as “first,” or “second” and so on.

It seems you only discussed limitations but not the strengths. What is the worth of studying without any strengths? Please discuss strengths as well.

Also, future proposition is too short. You may integrate this section under conclusions. Elaborate on your recommendation for future research. Also, write on the future directions for researchers, social workers, clinicians and policymakers.

**Answer:** We sincerely thank you for your constructive feedback. The following are our individual responses to the concerns raised: As a response to "Limitations and Strengths "in response, we have revised the section heading to "Limitations and Strengths" as suggested, ensuring a balanced discussion of both aspects. The transitional terms (e.g., "firstly," "secondly") have been replaced with a more formal structure (e.g., "First," "Second)(Lines303, 310-329). As a response to "Strengths of the Study "in response, a new paragraph has been added to highlight the study’s strengths(Lines304-310).

**References**

The references still show inconsistencies with the PLOS ONE journal submission guidelines. Please review them carefully and make the necessary corrections to ensure full compliance.

Ensure that all formatting adhere to the submission guidelines and are consistent throughout the manuscript.

**Answer:**Thank you for your meticulous review. We sincerely appreciate you bringing the reference formatting issues to our attention. We have thoroughly revised all references according to PLOS ONE's current guidelines.

**Reviewer #5:**

The manuscript investigates the mediating role of handgrip strength in the association between anemia and sleep disturbances among older adults in China by using CHARLS 2015 data. However, authors should clarify:

**Question 1:** Why iron status was not available and how its absence might limit interpretation.

**Answer:**

We are grateful to the reviewers for their profound comments on the iron state. We admit that the iron state data cannot be obtained in CHARLS 2015. Therefore, We are unable to determine whether the observed anemia cases are iron deficiency anemia or other types of anemia. We are unable to examine the potential dose-response relationship between iron status and handgrip strength or sleep disturbance. The mediating effect of handgrip strength may vary between iron deficiency anemia and other types of anemia. In future research, we will study iron deficiency anemia to better understand these relationships.

**Question 2:** How multicollinearity was assessed among anemia, grip strength, chronic conditions.

**Answer:** We appreciate your insightful suggestions. As you noted, we have stated in the Statistical Methods section that "multicollinearity was assessed using the Variance Inflation Factor (VIF) method, with a VIF of 5 or higher indicating the presence of multicollinearity." Furthermore, it should be noted that none of the variables exhibited multicollinearity**(Lines 144-147 and Supplementary Table 2)**.

**Question 3:** Whether the logistic regression models included survey weights or clustering corrections given complex sampling design.

**Answer:** We sincerely thank you for your profound opinions on the handling of complex sampling designs in CHARLS. CHARLS employs a multistage probability sampling design with unequal selection probabilities. In our analysis, we did not incorporate the provided survey weights to account for this design. We thank the reviewer for highlighting this important methodological consideration. In a future study, we would like to examine this issue.

**Question 4:** Whether interaction terms in subgroup analyses were statistically tested, if so, include p-values for interaction.

**Answer:** We appreciate your insightful comments on the interaction term tests in our subgroup analysis. In our study, we conducted a subgroup analysis to explore the impact of key variables on potential effects and performed statistical tests on the interaction terms**(**Supplementary Table 4 and 5).

**Question 5:** The methodological details such as variable coding, weighting, handling of missing data. The correlation coefficients in Table 3 are statistically significant but weak in magnitude like r = 0.04 so one should be careful in interpreting practical relevance. Provide more interpretative commentary in the main text for Figures.Also, tables may be placed in main text.Discuss whether sleep disturbance assessment via a single-item self-report question might underestimate sleep problems. The interpretation of handgrip strength should be presented with caution and may be tested with longitudinal data in future research.

**Answer:** We sincerely appreciate the reviewer’s thoughtful feedback and constructive suggestions, which have significantly improved the clarity and rigor of our manuscript.Below are our point-by-point responses: in response to the question "Interpretation of Weak Correlations". We agree that while correlations are statistically significant, their weak magnitude (e.g., r = 0.04) limits clinical interpretability. We now emphasize this in the Discussion Section(Lines 261-265). Inresponse to the question "Enhanced Commentary for Figures". We have refined the description of mediation analysis in the methods section and added confidence interval (95% ci) of the indirect effect in Figure 2 (Line 512). In response to the question "Single-Item Sleep Assessment Limitation". Thank you for your valuable suggestion. We have revised the relevant content in the discussion section (Lines 277-278).
