# Supplementary material for: The relationship between anemia and sleep disturbances among older Chinese adults: The mediating role of handgrip strength
Source: PLoS One. 2025 Oct 9;20(10):e0333673. doi: 10.1371/journal.pone.0333673 (PMC12510644; doi:10.1371/journal.pone.0333673)
Supplement: Attachment — Submitted filename: Response_to_Reviewers_auresp_2.doc [file pone.0333673.s011.doc]

**Response to Editor**

Dear Dr. Qian Wu

Thank you very much for your letter and the comments about our paper**“The Relationship between anemia and sleep disturbances among Older Adults in China: The Mediating Role of handgrip strength (Manuscript ID: 517084bef51b020d)”**. According to your suggestion, we have verified all cited references against PubMed databases and confirmed that none of the cited papers have been retracted.

After carefully studying the reviewers' comments and advice, we have made corresponding changes to the paper. Revised sections are identified with red text in the paper.

There are no conflicts of interest regarding this work. All authors have read the revised manuscript and approved its submission to the **PLOS ONE**. Please do not hesitate to contact us if we can be of any further assistance.

Thank you and best regards.

Yours Sincerely

Xiaojiang Zhao

E-mail: [zhaoxiaojiang2010@163.com](mailto:zhaoxiaojiang2010@163.com)

**Response to Reviewers**

Dear Reviewers.

Thank you very much for your letter and the comments about our paper **“The Relationship between anemia and sleep disturbances among Older Adults in China: The Mediating Role of handgrip strength (Manuscript ID: 517084bef51b020d)”**. After carefully studying the reviewers' comments and advice, we have made corresponding changes to the paper. Revised sections are identified with red text in the paper.

There are no conflicts of interest regarding this work. All authors have read the revised manuscript and approved its submission to the **PLOS ONE**. Please do not hesitate to contact us if we can be of any further assistance.

Thank you and best regards.

Yours Sincerely

Xiaojiang Zhao

mail: zhaoxiaojiang2010@163.com

****Response to Reviewer #1****

We sincerely appreciate the reviewer's thoughtful feedback and constructive suggestions. We have carefully addressed each point as detailed below, with all changes tracked in the revised manuscript (highlighted in yellow for ease of review).

Dear Authors, Thank you again for your diligent revisions. Your softened causal language, expanded biological discussion, and clearer hypothesis framing have greatly improved the manuscript. I recommend acceptance pending a few minor edits and clarifications, especially around distinguishing mediation from confounding and correcting a mis-applied citation. Please address the points below—organized by line number:

**Question 1:** Line 51–52 Issue: Wording implies anemia is a subtype of “nutritional deficiencies,” and Ref. 15 does not address anemia.

Fix: Either remove “anemia” from this clause or replace it with a citation specifically linking anemia to sleep disturbances.

**Answer:** We sincerely appreciate your careful reading. To avoid overgeneralizing the relationship between nutritional deficiencies and sleep disturbances, we have removed “anemia” from the clause in lines 51, 52. Thank you for helping us improve the precision of our manuscript.

**Question 2:** Lines 106–108 Issue: WHO hemoglobin cutoffs (<12.0 g/dL women; <13.0 g/dL men) omit age-related declines.

Fix: Add a brief note in Methods acknowledging this limitation (and, if feasible, mention a sensitivity analysis with age-specific ranges).

**Answer:** As suggested, we have added the following acknowledgment in the Methods section (Lines 114, 115):"While WHO cutoffs provide standardized criteria, we acknowledge these do not account for age-related hemoglobin declines."

**Question 3:** Lines 203–205 Issue: Typo “sleep disturbanced breathing.”

Fix: Correct to “sleep-disordered breathing. ”

**Answer:** Thank you for pointing out this typographical error. We sincerely apologize for the oversight. As suggested, we have corrected “sleep disturbanced breathing” to “sleep-disordered breathing” in Line 212. This revision ensures accuracy and consistency with standard medical terminology.

**Question 4:** Lines 206–207 Issue: “Lower anemia” is imprecise.

Fix: Change to “lower hemoglobin levels” or “milder anemia. ”

**Answer:** Thank you for your insightful comment. We agree that the term "Lower anemia" is imprecise. As suggested, we have revised the text to "lower hemoglobin levels"(Line214).

**Question 5:** Lines 195–199 & 241–248 Issue: Some definitive causal verbs remain (“mediates,” “could alleviate sleep disturbances”).

Fix: Replace with circumspect phrasing (“may mediate,” “is associated with,” “could reflect”).

**Answer:** Thank you for your valuable feedback. As suggested, we have revised the language to reflect a more cautious interpretation(Lines239, 256). These changes align with the observational nature of our study and ensure consistency with the available evidence. We appreciate your attention to this important nuance.

**Question 6:** Numbering and Cross-References Issue: Section/table labels (e.g., S4 vs. S5) and in-text citations sometimes mismatch.

Fix: Ensure every cited figure/table corresponds to the correct label.

**Answer:** Thank you for your careful review. We have thoroughly checked all section/table labels (e.g., S4, S5) and in-text citations as suggested, and confirmed Confirm that all references in the current version correspond correctly to the tags.If we missed any inconsistencies, please kindly point out the specific instances, and we will promptly correct them. We greatly appreciate your attention to detail.

**Question 7:** Clarify Mediation vs. Confounding Several passages conflate mediator pathways with confounding influences. Please revise as follows: Iron-Deficiency Mechanism (Lines 206–217): Label body iron status as a confounder, not a mediator, or move to Limitations, noting ferritin wasn’t measured. Inflammatory Pathways (Lines 248–252): Recast inflammation as a shared cause of both reduced grip strength and poor sleep, or explicitly justify how it transmits effects through grip strength. Bidirectional Interplay (Lines 246–249): Choose one primary direction (grip → sleep) for mediation, and present the reverse (sleep → grip) as an alternative hypothesis or effect modifier—not part of the same mediation pathway.

**Answer:** We sincerely thank the reviewers for their insightful feedback on our analysis. We have made the following revisions to the manuscript to address these issues:

As a response to "Clarify Mediation vs. Confounding". In response, we have revised the text to explicitly frame body iron status as a potential confounder rather than a mediator(Lines 215-217).

As a response to "Inflammatory pathway". In response, we agree that the temporal relationship between inflammation, grip strength, and sleep requires further elaboration. Just as suggested, we have modified the text(Lines 264-268).

As a response to "Bidirectional Interplay". In response, We sincerely appreciate the reviewer's insightful comment regarding the bidirectional interplay between grip strength and sleep quality. Upon careful consideration, we agree that presenting both directions within the same mediation framework could create conceptual ambiguity. In response: We have removed the bidirectional Interplay (original Lines 260-265) to maintain methodological clarity. This modification strengthens the paper's focus while preserving scientific rigor. We thank the reviewer for prompting this important clarification.

**Question 8:** Muscle Fatigue “Recovery” Mechanism (Lines 249–252)

Current text: “Grip strength reflects overall muscle function, and the anemia-induced weakness can cause increased daytime fatigue, which may necessitate more intense recovery during sleep and potentially disrupt sleep patterns[47].”

Issues: The cited Debevec et al. study examines normobaric hypoxia and bed-rest muscle wasting—environmental oxygen deprivation under inactivity—not anemia-induced hypoxia or sleep architecture. Anemia-related hypoxia co-occurs with other factors (e.g., micronutrient deficiencies, inflammation), making it not equivalent to pure environmental hypoxia.

Fix: Either Provide a sleep-specific citation that directly links anemia-induced muscle fatigue or elevated metabolic demand to disrupted sleep stages, or Flag as speculative, for example: “This remains hypothetical—while environmental hypoxia can exacerbate inactivity-related muscle wasting[Debevec et al. 2018], anemia-related hypoxia (often accompanied by micronutrient deficiencies and inflammation) may not have the same effects on sleep architecture, and this pathway requires direct study.”

Once these edits and clarifications are made, the manuscript will present a coherent, well-justified narrative—appropriately cautious about causality and clear in its distinction between mediators and confounders.

**Answer:** We sincerely appreciate the reviewer's insightful critique regarding the speculative link between anemia-induced muscle fatigue and sleep disruption. We fully agree that the cited study on normobaric hypoxia (Debevec et al.) does not directly address anemia-related pathways, and we have revised the text to better reflect this distinction. Following the reviewer's second suggested approach, we have: Removed the original speculative statement linking anemia to sleep disruption via muscle recovery. Replaced it with a more cautious formulation that explicitly acknowledges the knowledge gap(Lines 258-263). We believe this revision better aligns with the available evidence while preserving the conceptual framework for future research. Thank you for highlighting this important nuance.

****Response to Reviewer #4****

**Question 1 :** I do, however, have a few remaining suggestions that were not fully addressed in your previous response. While my training in Epidemiology typically advises against using cross-sectional data for mediation analysis, I understand that such approaches are commonly employed in the literature to explore associations. Given this context, I would recommend that you explicitly and critically acknowledge this methodological limitation in your study.

**Answer:** We sincerely appreciate the reviewer's thoughtful comments regarding the methodological considerations of mediation analysis with cross-sectional data. We fully acknowledge the limitations highlighted by the reviewer. To address this limitation, we have: Added a dedicated subsection in the Discussion. The limitations of the research methods were clearly and critically acknowledged. And point out the research direction we aim for in the longitudinal study (Lines 285-290).

**Question 2 :** Abstract

Methods

Since you do not entail what specific inclusion and exclusion criteria were applied in the abstract, you may just say that 6,057 is your analytic sample. It is by default understood that the analytic sample is a result of certain operation and treatments to the original dataset.

**Answer:** We appreciate the reviewer’s constructive suggestions. Below are our responses:

As a response to "Analytic sample". In response, we have revised the abstract to state “Following the application of specific inclusion and exclusion criteria, a total of 6,057 Chinese adults aged 60 and above were finally selected as the analysis samples. ” without detailing inclusion/exclusion criteria to save space(Lines 17,18).

As a response to "Variable categories". In response, we have simplified the description of variables in brackets (e.g., sleep [yes/no], anemia [present/absent]) as recommended(Lines 18-20).

As a response to "Regression terminology". In response, we agree that both terms are technically correct. For clarity, we will use “multivariable logistic regression” in the manuscript to emphasize the adjustment for multiple covariates. We thank the reviewer for these thoughtful comments, which have improved the conciseness of our abstract.

**Question 3 :** Introduction

Please check the PLOS One formatting guidelines if there should be space before [] sign used for in-text citation. If yes, make changes throughout the manuscript.

**Answer:** We appreciate the reviewer's careful attention to citation formatting. According to PLOS ONE guidelines, we have now ensured that all in-text citations (e.g., [1]) are preceded by a space throughout the manuscript. All instances have been corrected in the revised version.

**Question 4:** <1st paragraph> While I appreciate the operationalization of "sleep disturbance" in the Methods section, I would respectfully suggest that it might be beneficial to introduce and contextualize the term earlier in the manuscript based on existing literature, ideally in the Introduction. Providing a brief overview of how "sleep disturbance" is defined in the scientific literature—or acknowledging the lack of a standardized definition—could help readers better understand the scope and relevance of your study. Given that "sleep disturbance" can encompass a range of issues such as insomnia, obstructive sleep apnea, and excessive daytime sleepiness, clarifying what is meant in your study from the outset could enhance the clarity and impact of your work. The operational definition fits well under the methods section.

**Answer:** As you recommended, we have now added a concise definition of "sleep disturbance" in the Introduction section(Lines 38,39). We believe these additions enhance the manuscript’s readability and theoretical grounding. Thank you again for your constructive comment. Please let us know if further clarifications would be helpful.

**Question 5 :** <4th paragraph> Apologies if I may have overlooked it, but I did not clearly see the theoretical foundation referenced in lines 75–78. If it is present, it may benefit from clearer articulation. Additionally, as a general convention, the theoretical framework is typically introduced before the last paragraph in the introduction section where you describe the research objective. Also, ideally the conceptual framework positioned after the research objectives and before the Methods section. Presenting it there can help anchor the study's rationale and guide readers through the analytical approach more effectively. I would recommend using conceptual framework than DAGS since your study is cross-sectional.

**Answer:** We sincerely appreciate the reviewer’s thoughtful suggestions to improve the manuscript’s clarity and structure. As suggested, we added the "conceptual framework" after the research objective and before the Methods(Lines 78-80,S1 Fig). We hope the revised manuscript addresses the reviewer’s concerns effectively.

**Question 6:** Methods <1st paragraph> If CHARLES is a longitudinal study, not a part of longitudinal study, then please correct the statement where it means that CHARLES is a part of a longitudinal study.

**Answer:** Thank you for your careful reading and valuable feedback.We sincerely appreciate your attention to detail, which has helped us improve the accuracy of our manuscript. We apologize for any confusion caused by the original phrasing. To clarify: CHARLS is a longitudinal study. We have revised the Methods section (Lines 83, 84) to explicitly state: "CHARLS is a longitudinal survey that represents the national population of Chinese individuals aged 45 and above living in communities.". We thank the reviewer for highlighting this important clarification. The revised text now accurately reflects the study design. Please let us know if further adjustments are needed.

**Question 7:** Given that the CHARLS dataset is longitudinal, your decision to rely solely on Wave 3 for a cross-sectional analysis warrants a clear and well-reasoned justification—particularly in light of your acknowledgment that research on the causal relationship between anemia and sleep disturbance remains limited. What distinguishes your cross-sectional study from existing ones that have already explored this association? Does your work offer new insights, or does it largely replicate previously established findings? Additionally, it is important to address the limitations inherent in using cross-sectional data to test mediating relationships. Such analyses are generally viewed as exploratory rather than confirmatory, due to the inability to establish temporal ordering and causal direction. Given you already have access to longitudinal data, why motivates you to prefer a cross-sectional analysis? Please, clarify the rationale behind focusing exclusively on a single wave for establishing the validity and contribution of your study.

Your response was:
Thank you for the important questions you raised about our research methods. Regarding our decision to use CHARLS Wave 3 data for cross-sectional analysis rather than longitudinal analysis, we understand your concerns and are willing to explain our considerations in detail. First of all, our research builds on previous literature on the association between anemia and sleep disturbances, but we focused on older Chinese adults and explored the mediating role of handgrip strength in the association between anemia and sleep disturbances. Secondly, the response rate and data quality of Wave 3 are the most ideal among all waves of CHARLS. In the future, we will follow your suggestion and conduct research using the complete longitudinal
data of CHARLS.
While mediation analysis can be conducted using cross-sectional data for exploratory purposes, it is ideally suited to longitudinal designs due to the need for temporal ordering to support causal inference. Given that CHARLS is a longitudinal dataset, it would be helpful to clarify whether the key variables were available across multiple waves. If not, this would justify the use of cross-sectional data. However, the rationale provided—based on response rate and data quality—does not fully address the choice to forgo a longitudinal approach. If you were only exploring the relationship without including the mediation, cross-sectional dataset would be fine. But, since you included mediation, I recommend explicitly acknowledging this limitation in the manuscript, especially considering the availability of the variables of interest in multiple waves of the longitudinal data.
Also, you might consider revisiting the rationale on focusing older adults, as I found several pre-existing observational studies exploring this relationship in Chinese adult and older adult population. I included some of them below. Your rationale should justify what different or important exploration your study is contributing in light of pre-existing literature on this topic. These are crucial to ensure rigor of the study.
Cheng C, Chen X, Zhang L, et al. A Risk Correlative Model for Sleep Disorders in Chinese Older Adults Based on Blood Micronutrient Levels: A Matched Case-Control Study. Nutrients. 2024;16(19):3306. Published 2024 Sep 29. doi:10.3390/nu16193306
Neumann SN, Li JJ, Yuan XD, et al. Anemia and insomnia: a cross-sectional study and meta-analysis. Chinese Medical Journal. 2021;134(6):675-681. doi:10.1097/CM9.0000000000001306
Kara O, Elibol T, Koc Okudur S, Smith L, Soysal P. Associations between anemia and insomnia or excessive daytime sleepiness in older adults. Acta Clinica Belgica. 2023;78(3):223-228. doi:10.1080/17843286.2022.2116895

**Answer:** Thank you for your thoughtful comments and for bringing these relevant studies to our attention. We appreciate the opportunity to clarify how our study builds upon and extends the existing literature on anemia and sleep disturbances in older Chinese adults.

Key distinctions and contributions of our study:

**Novel mediating mechanism:** While previous studies have established associations between anemia and sleep disturbances, our study uniquely examines handgrip strength as a potential mediator in this relationship. This physiological pathway has not been thoroughly investigated in the existing literature you cited.

**Nationally representative sample:** The CHARLS data provides a unique opportunity to examine these relationships in a large, nationally representative sample of Chinese older adults, enhancing the generalizability of findings compared to regional or clinical samples used in some previous studies.

**Clinical implications:** By identifying handgrip strength as a potential mediator, our findings could inform more targeted interventions (e.g., combining anemia treatment with resistance training) compared to studies examining only the direct anemia-sleep relationship.

Thank you again for helping us strengthen the manuscript's theoretical foundation and clinical relevance.

**Question 8:** Statistical Methods

Please add a citation which you refer to using VIF cut-off point of 5 or above.

**Answer:** Thank you for your constructive comment. We added citations to support the VIF threshold in the Statistical Methods section as suggested by the reviewer (Line 146, 147).
