# Supplementary material for: The relationship between anemia and sleep disturbances among older Chinese adults: The mediating role of handgrip strength
Source: PLoS One. 2025 Oct 9;20(10):e0333673. doi: 10.1371/journal.pone.0333673 (PMC12510644; doi:10.1371/journal.pone.0333673)
Supplement: Attachment — Submitted filename: Response_to_Reviewers_auresp_3.doc [file pone.0333673.s012.doc]

**Response to Editor**

Dear Dr. Qian Wu

Thank you very much for your letter and the comments about our paper**“The Relationship between anemia and sleep disturbances among Older Adults in China: The Mediating Role of handgrip strength (Manuscript ID: 517084bef51b020d)”**. According to your suggestion, we have verified all cited references against PubMed databases and confirmed that none of the cited papers have been retracted.

After carefully studying the reviewers' comments and advice, we have made corresponding changes to the paper. Revised sections are identified with red text in the paper.

There are no conflicts of interest regarding this work. All authors have read the revised manuscript and approved its submission to the **PLOS ONE**. Please do not hesitate to contact us if we can be of any further assistance.

Thank you and best regards.

Yours Sincerely

Xiaojiang Zhao

E-mail: [zhaoxiaojiang2010@163.com](mailto:zhaoxiaojiang2010@163.com)

**Response to **Reviewer #1****

**Dear Dr. Carmine TC**

Thank you very much for your letter and the comments about our paper **“The Relationship between anemia and sleep disturbances among Older Adults in China: The Mediating Role of handgrip strength (Manuscript ID: 517084bef51b020d)”**. After carefully studying the reviewer' comments and advice, we have made corresponding changes to the paper. Revised sections are identified with red text in the paper.

There are no conflicts of interest regarding this work. All authors have read the revised manuscript and approved its submission to the **PLOS ONE**. Please do not hesitate to contact us if we can be of any further assistance.

Thank you and best regards.

Yours Sincerely

Xiaojiang Zhao

mail: zhaoxiaojiang2010@163.com

****Response to Reviewer #1****

**Question：** Recommendation for Final Revision:

To bring this manuscript to a publishable standard, I propose a clear structural solution that resolves these logical issues and enhances its scientific rigor:

Retain Mechanism 1 ("Muscle fatigue") as the sole hypothesized mediational pathway, continuing to emphasize its speculative nature.

Remove the "Inflammatory pathways" and "Energy metabolism" sections from the list of mediational mechanisms.

Instead, create a new, separate paragraph in the Discussion, for instance, under the heading "Alternative Interpretations and Potential Confounding." In this new paragraph, place the argument regarding inflammatory pathways, explicitly framing it as a major potential confounder that could also explain the observed associations. Also in this paragraph, discuss how "impaired energy metabolism" could represent a direct physiological effect of anemia on sleep, operating in parallel with the statistical mediation observed(Lines 265-274).

**Answer:** We sincerely appreciate your profound insights and constructive suggestions regarding the manuscript. Regarding your valuable suggestions on the analysis of the intermediary mechanism, we have carefully made the necessary revisions.

(1). Rationality of structural adjustment

We fully share your core concern about strengthening logical rigor. In the revised version, we have clearly designated the "muscle fatigue" mechanism as the primary assumed intermediary pathway.

(2). Maintaining the academic considerations of other mechanisms

After thorough discussion within the team, we have decided to substantially rewrite the "inflammatory pathway" and "energy metabolism" sections rather than delete them. Based on theoretical integrity: The existing evidence indicates that these pathways have an independent association with anemia-sleep.

These modifications not only maintain the scientific rigor you suggested, but also avoid the potential omission of biological mechanisms due to oversimplification. We believe that the current version more fully reflects the complexity of the issue and is in line with the best practices of causal inference.

The attached revised draft has highlighted all the corresponding changes in red. We sincerely request that you review these adjustments to see if they appropriately address your concerns. We are fully open to further discussion or modifications on this matter.

Once again, we would like to express our gratitude for the valuable time and professional guidance you have provided in enhancing the quality of this article.
