# Supplementary material for: The relationship between anemia and sleep disturbances among older Chinese adults: The mediating role of handgrip strength
Source: PLoS One. 2025 Oct 9;20(10):e0333673. doi: 10.1371/journal.pone.0333673 (PMC12510644; doi:10.1371/journal.pone.0333673)
Supplement: Attachment — Submitted filename: Response_to_Reviewers_auresp_4.doc [file pone.0333673.s013.doc]

**Response to Editor**

Dear Dr. Qian Wu

Thank you very much for your letter and the comments about our paper**“The Relationship between anemia and sleep disturbances among Older Adults in China: The Mediating Role of handgrip strength (Manuscript ID: 517084bef51b020d)”**. According to your suggestion, we have verified all cited references against PubMed databases and confirmed that none of the cited papers have been retracted.

After carefully studying the reviewers' comments and advice, we have made corresponding changes to the paper. Revised sections are identified with red text in the paper.

There are no conflicts of interest regarding this work. All authors have read the revised manuscript and approved its submission to the **PLOS ONE**. Please do not hesitate to contact us if we can be of any further assistance.

Thank you and best regards.

Yours Sincerely

Xiaojiang Zhao

E-mail: [zhaoxiaojiang2010@163.com](mailto:zhaoxiaojiang2010@163.com)

**Response to **Reviewer #1****

**Dear Dr. Carmine TC**

Thank you very much for your letter and the comments about our paper **“The Relationship between anemia and sleep disturbances among Older Adults in China: The Mediating Role of handgrip strength (Manuscript ID: 517084bef51b020d)”**. After carefully studying the reviewer' comments and advice, we have made corresponding changes to the paper. Revised sections are identified with red text in the paper.

There are no conflicts of interest regarding this work. All authors have read the revised manuscript and approved its submission to the **PLOS ONE**. Please do not hesitate to contact us if we can be of any further assistance.

Thank you and best regards.

Yours Sincerely

Xiaojiang Zhao

mail: zhaoxiaojiang2010@163.com

****Response to Reviewer #1****

**Question:** Thank you for the revised manuscript and for the thoughtful response letter. I am recommending Minor Revision because I believe the final changes required are straightforward to implement. However, this recommendation is strictly conditional on resolving the core conceptual issue that, despite the recent revisions, has persisted from my previous review.

In your response letter, you articulated your rationale for the current approach, citing a desire to preserve "theoretical integrity" and "avoid the potential omission of biological mechanisms." I fully agree that these mechanisms are important. The crucial point that was missed in this revision, however, is that these mechanisms must be framed with correct causal terminology to ensure the paper's logical integrity.

The central issue remains: the manuscript continues to conflate mediation with confounding. For example, describing how inflammation affects both grip strength and sleep independently is a description of confounding, not mediation. Presenting it as a mediating pathway is a significant logical flaw that undermines the paper's main conclusion.

To resolve this persistent issue once and for all, I must insist on a specific structural change. This is not a suggestion, but a requirement for my recommendation of acceptance.

Required Action:

Clearly designate "Muscle fatigue and recovery" as the primary hypothetical mediating pathway.

Create a new, separate paragraph with the heading: "Alternative Explanations and Potential Confounding".

Move your important discussions of "inflammatory pathways" and "energy metabolism" into this new paragraph. You must then reframe them using precise causal language.

For example:

For Inflammation: State that it may act as a common cause or confounder.

For Energy Metabolism: Explain that it could represent a confounding pathway or a direct effect of anemia.

Why This Non-Negotiable Revision is Necessary:

It Corrects the Persistent Logical Flaw: This structure finally distinguishes between your proposed mediation hypothesis and its most likely alternatives.

It Aligns with Scientific Rigor: Correctly labeling potential confounders is a sign of a robust scientific argument and is a best practice in causal inference.

It Provides a Clear Path to Acceptance: This offers a simple, actionable solution to the only major issue remaining in an otherwise improved manuscript.

I trust that the authors will now understand the necessity of this change. I look forward to reviewing a final version that incorporates these specific revisions and will be pleased to recommend acceptance at that time.

**Answer:** We sincerely thank you for your comments on the "intermediary role and confounding factors" issue in our manuscript as well as for your constructive suggestions. Your insights have been invaluable in strengthening the logical rigor and clarity of our work. We have carefully implemented all the requested changes as outlined below:

1). As you rightly pointed out, we have: Clearly designated "Muscle fatigue and recovery" as the primary mediating pathway in the revised manuscript (Lines 258,259).

2).Created a new subsection titled "Alternative Explanations and Potential Confounding" (Lines 266,278). Where we now discuss: Inflammatory pathways as potential common causes/confounders. Energy metabolism as a potential confounding pathway.

Once again, we would like to express our gratitude for the valuable time and professional guidance you have provided in enhancing the quality of this article. Please let us know if further adjustments are needed.
